# Chronic stress disrupts hepatic homeostasis and accelerates liver cancer progression through ADRB2 signaling

**DOI:** 10.1126/sciadv.aec0825

**Published:** 2026-05-01

**Authors:** Huimin Qin, Liuwei Chu, Xiaying Zheng, Huimei Zhang, Yue Lan, Ji Hu, Lu Li

**Affiliations:** ^1^ School of Life Science and Technology, ShanghaiTech University, Shanghai 201210, China.; ^2^ Precision Research Center for Refractory Diseases, Shanghai Jiao Tong University Pioneer Research Institute for Molecular and Cell Therapies, Shanghai General Hospital, Shanghai Jiao Tong University School of Medicine, Shanghai 201620, China.; ^3^ State Key Laboratory of Innovative Immunotherapy, School of Pharmaceutical Sciences, Shanghai Jiao Tong University, Shanghai 200240, China.; ^4^ Department of Rehabilitation Medicine, School of Medicine, the Second Affiliated Hospital of South China University of Technology (Guangzhou First People’s Hospital), Guangzhou, Guangdong 510180, China.

## Abstract

Chronic stress has been implicated in the dysregulation of immunological, neurochemical, and endocrinological functions, yet its impact on hepatic homeostasis and liver carcinogenesis remains elusive. In this study, by using single-nucleus RNA sequencing and histopathological evaluation, we demonstrated that chronic stress induced profound hepatic dysfunction. Notably, using orthotopic murine models of liver cancer, we further found that chronic stress substantially accelerated tumor progression. Mechanistically, we identified β2-adrenergic receptor (ADRB2) signaling as the pivotal molecular pathway driving stress-accelerated cancer progression, which was in line with the poor clinical outcomes observed in patients with cancer exhibiting enhanced adrenergic signaling within tumors. Collectively, our study provides compelling evidence that chronic stress compromises hepatic homeostasis and accelerates liver cancer progression via ADRB2 activation, highlighting the therapeutic potential of targeting this pathway and the clinical importance of stress management in hepatic disorders.

## INTRODUCTION

Chronic stress, a pervasive yet underappreciated contributor to global disease burden, has been documented to dysregulate cross-system physiological networks, including immune surveillance, neuroendocrine signaling, and metabolic regulation. Accumulating evidence demonstrates that chronic stress serves as a key pathogenic driver of tissue dysfunction and contributes to the development of multiple systemic disorders, notably diabetes mellitus and cardiovascular diseases (*1*). The liver, as the body’s central metabolic processing hub, occupies a critical position in mediating stress-induced systemic perturbations. Nevertheless, mechanistic investigations into stress-mediated hepatic pathophysiology remain unexpectedly limited. A comprehensive understanding of the impact of chronic stress on hepatic homeostasis is imperative for the effective prevention of hepatic disorders.

Patients with cancer represent a population particularly vulnerable to the detrimental effects of chronic stress (*2*, *3*). Clinical and preclinical studies have linked chronic stress exposure to accelerated progression across multiple malignancies (*4*, *5*), including lung cancer (*6*), pancreatic cancer (*7*), prostate cancer (*8*), and ovarian cancer (*9*). Emerging evidence suggests that stress systemically facilitates cancer progression through modulating most hallmarks of cancer (*4*, *10*). Critically, however, the direct cellular targets and precise molecular mechanisms underlying chronic stress–induced tumor progression demonstrate remarkable tissue specificity. Whereas adrenergic activation has been mechanistically linked to enhanced ovarian carcinoma progression (*9*), stress-induced neuroendocrine activation exhibits a negligible effect on the growth of primary breast tumor (*11*). This tumor type–dependent divergence may be mediated by differential expression patterns of stress hormone receptors, particularly adrenergic receptors (ARs) that gatekeep oncogenic stress responsiveness, among malignant cells, immune populations, and stromal compartments within distinct tumor microenvironments (*4*, *12*, *13*).

Liver cancer, the sixth most prevalent malignancy globally, exhibits a mortality rate ranking third and a 5-year survival rate of merely 18% (*14*–*17*). Hepatocellular carcinoma (HCC) is the dominant form of primary liver cancer (~80% of cases), followed by intrahepatic cholangiocarcinoma (~15%) (*17*). This disease is frequently diagnosed at an advanced stage when therapeutic options are severely limited. To date, extensive research efforts have focused on the dysregulation of oncogenes and tumor suppressors in tumor cells or the immune microenvironment. Anatomically, the liver receives dense sympathetic innervation (*18*) and is continuously exposed to systemic stress signals in the bloodstream. Although epidemiological observations have identified psychological distress in some patients with liver cancer (*19*), whether and by what molecular mechanisms chronic stress regulates hepatocarcinogenesis remains largely unexplored. This knowledge gap persists due in large part to the prevalent reliance on subcutaneous xenograft models and acute, nonphysiological catecholamine administration, which fail to recapitulate the spatiotemporal dynamics of neuroendocrine signaling within the native liver microenvironment (*20*–*23*). Therefore, establishing a model that integrates an orthotopic tumor within a chronically stressed host is essential to definitively elucidate the pathophysiological role and mechanisms of chronic stress in liver cancer progression.

In this study, we performed a comprehensive investigation into the pathophysiological effects of chronic stress on liver disorders. Here, single-cell resolution transcriptome profiling, in conjunction with blood biochemistry and histopathological evaluation, was used to systematically characterize the effects of chronic stress on hepatic homeostasis and liver malignancies. Through the well-established chronic stress paradigms and orthotopic murine liver cancer models, we identified β2-adrenergic receptor (ADRB2)-mediated adrenergic signaling as a central mechanistic link between chronic stress and tumor progression. Furthermore, the correlation between ADRB2 signaling and cancer progression was validated in human clinical datasets. The study establishes a brain-liver axis paradigm, demonstrating how systemic physiological states can directly reprogram peripheral organ function, and provides compelling evidence for stress management in the prevention and therapeutic intervention of liver malignancies.

## RESULTS

### Chronic stress impairs hepatic homeostasis

**Fig. 1.**
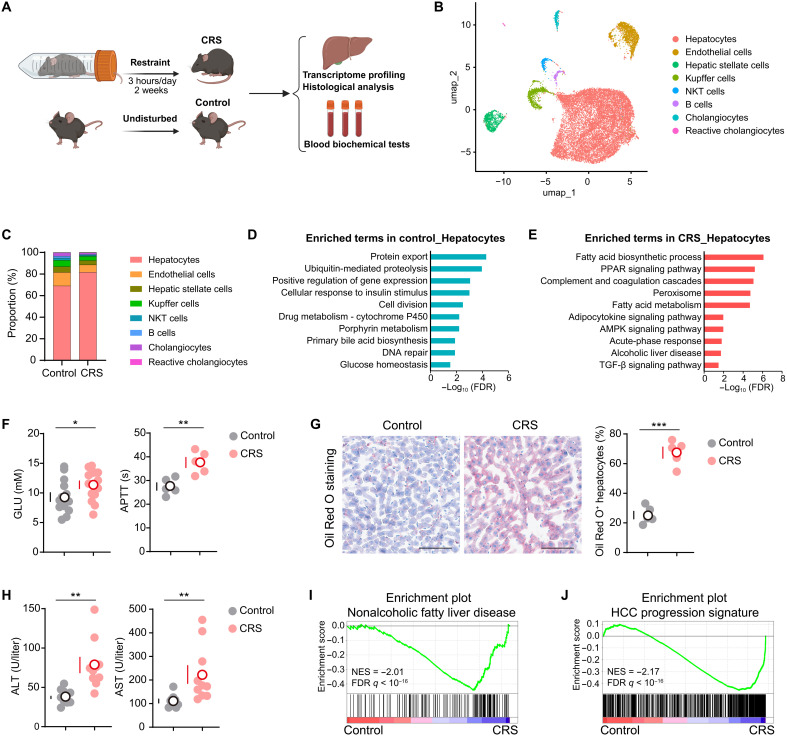
Chronic stress impairs hepatic homeostasis. (**A**) Schematic view showed that mice subjected to 2-week CRS for 3 hours/day (CRS group) or left undisturbed (control group) were scheduled for transcriptome profiling of the livers, histological analysis of the livers, and blood biochemical tests. Created in BioRender. Li, L. (2026) https://BioRender.com/ya0qhak. (**B**) UMAP (Uniform Manifold Approximation and Projection) visualization of all liver cells in the control group and CRS group generated by 10× chromium protocol. Colors indicated cell subpopulations. (**C**) Cellular proportions of cell subpopulations. (**D** and **E**) Enriched Gene Ontology (GO) and Kyoto Encyclopedia of Genes and Genomes (KEGG) terms in control hepatocytes (D) or hepatocytes from the CRS group (E). (**F**) Blood glucose levels (control, *n* = 14; CRS, *n* = 15) and APTT (*n* = 5). (**G**) Representative images of Oil Red O staining of liver sections. The ratio of Oil Red O–stained hepatocytes was quantified (*n* = 5). (**H**) Serum ALT and AST levels (*n* = 10). (**I** and **J**) GSEA showed that the gene sets of NAFLD (I) and HCC progression signature genes (J) were enriched in CRS-exposed mouse livers when compared with control livers. Scale bars, 100 μm. Data are presented as the mean ± SEM; *P < 0.05, **P < 0.01, and ***P < 0.001, *t* test. FDR, false discovery rate; PPAR, peroxisome proliferator–activated receptor; AMPK, AMP-activated protein kinase; TGF-β, transforming growth factor–β; NES, normalized enrichment score.

To comprehensively delineate the effect of chronic stress on the liver, we performed transcriptome profiling, histopathological evaluation, and blood biochemistry tests (Fig. 1A). We used the well-established chronic restraint stress (CRS) paradigm, in which mice exhibit depression-like behaviors and recapitulate the stress-induced neuroendocrine dysregulation observed in humans (*24*, *25*). CRS-exposed mice exhibited elevated neuronal activation across multiple brain regions compared to control cohorts (fig. S1, A to I), concurrent with a robust reduction in body weight (fig. S1J), validating the efficacy of the stress paradigm.

Single-nucleus RNA sequencing (snRNA-seq) was conducted using the 10× Genomics Chromium platform. Using established cell type–specific marker genes, we identified eight major cell populations, including hepatocytes, endothelial cells, hepatic stellate cells, Kupffer cells, Natural Killer T (NKT) cells, B cells, cholangiocytes, and reactive cholangiocytes (Fig. 1B, and fig. S2, A to E). Comparative cellular composition analysis demonstrated a notable increase in hepatocytes proportion in the CRS group, accompanied by decreased frequencies of nonparenchymal cell (NPC) populations, particularly Kupffer cells (liver-resident macrophages) (Fig. 1C), which was validated at both mRNA and protein levels (fig. S2, F to I).

As the primary effector cells for liver function, hepatocytes were subjected to comprehensive transcriptomic profiling to delineate stress-induced molecular perturbations. Pathway enrichment analysis of differentially expressed genes (DEGs) demonstrated that control hepatocytes were enriched in gene sets related to fundamental hepatic processes, including protein export, proteolysis, cell division, drug metabolism, and glucose homeostasis (Fig. 1D). CRS-exposed hepatocytes exhibited robust activation of stress-responsive pathways, particularly fatty acid metabolism, alcoholic liver disease, peroxisome proliferator–activated receptor pathway, AMP-activated protein kinase pathway, transforming growth factor-β pathway, and coagulation (Fig. 1E), suggesting a molecular framework for stress-associated hepatic dysfunction. Notably, we found that chronic stress induced hyperglycemia in mice (Fig. 1F), which was primarily attributable to impaired hepatic glucose regulation (fig. S3, A to E). Activated partial thromboplastin time (APTT) was elevated in CRS-exposed mice (Fig. 1F), signifying impaired coagulation following chronic stress. Histopathological assessment demonstrated that chronic stress induced liver steatosis (Fig. 1G), the early harbingers of nonalcoholic fatty liver disease (NAFLD) (*26*). Moreover, clinical chemistry analyses of hepatic injury biomarkers revealed that alanine aminotransferase (ALT) and aspartate aminotransferase (AST) were increased in CRS-exposed mice (Fig. 1H).

To corroborate the above findings, we performed complementary bulk RNA-seq analysis to achieve greater transcriptomic coverage. Differential expression analysis identified that stress response genes, lipid metabolic genes, and oncogenic drivers were potently induced in CRS-exposed livers (fig. S3, F and G). Functional enrichment analysis demonstrated marked activation of disease-associated signatures, including NAFLD and HCC progression genes in CRS-exposed livers (Fig. 1, I and J; fig. S3H; and table S1). Collectively, these multimodal analyses demonstrate that chronic stress induces a cascade of molecular, cellular, and physiological alterations that disrupts hepatic homeostasis toward a protumorigenic molecular landscape.

### Chronic stress accelerates tumor progression in *Akt*;*Nras*-driven liver cancer

**Fig. 2.**
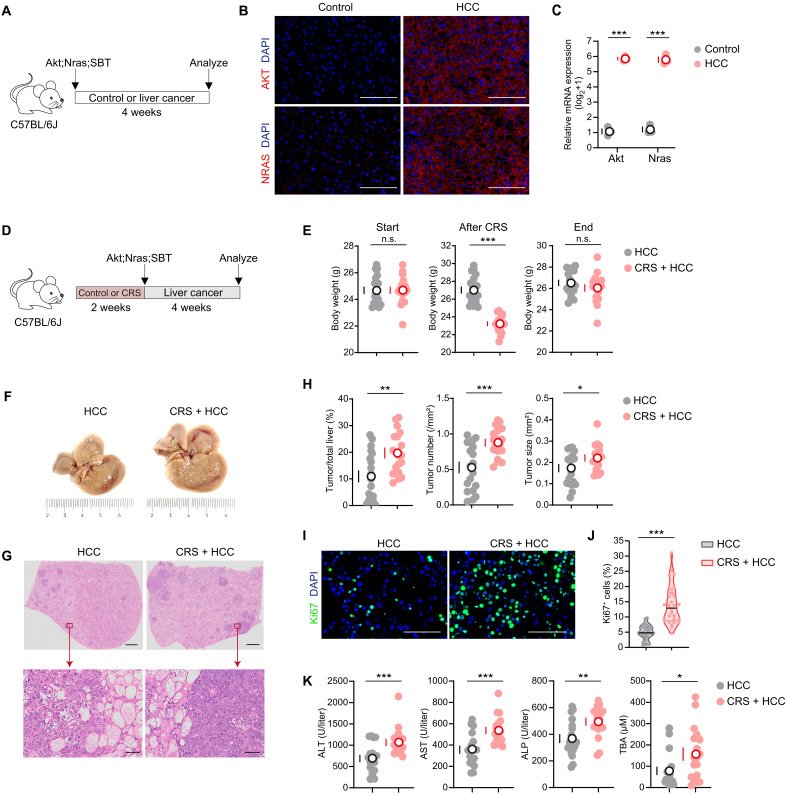
Chronic stress accelerates tumor progression in *Akt*;*Nras*-driven liver cancer. (**A**) Schematic view. Eight-week-old mice were induced liver cancer by HTVi of Akt;Nras;SBT. Mice left undisturbed served as controls. All mice were euthanized 4 weeks later to quantify the expression of *Akt* and *Nras* [(B) and (C)]. (**B**) Representative immunofluorescence images. (**C**) qPCR measured the mRNA levels of *Akt* and *Nras* (*n* = 3). Data were presented as log_2_(expression+1). (**D**) Schematic view of CRS treatment and tumor modeling. Eight-week-old mice were subjected to CRS or left undisturbed. After 2 weeks, all mice were HTVi of Akt;Nras;SBT to induce liver cancer. All mice were euthanized 4 weeks later to quantify the tumor formation [(E) to (K); HCC, *n* = 19; CRS + HCC, *n* = 18]. (**E**) Body weights at the start of the experiment, after 2-week CRS, and at the end of the experiment. (**F**) Representative liver images. (**G**) Representative hematoxylin and eosin (H&E) staining images of liver sections. (**H**) The ratios of tumor/total liver area, tumor number per square millimeter, and tumor size. (**I**) Immunofluorescent staining of the proliferation marker Ki67. (**J**) The ratio of Ki67^+^ cells (*n* = 6). The median was indicated. (**K**) Serum levels of ALT, AST, ALP, and TBA. Scale bars, 100 μm {except for [(G), top], 1 mm}. Except for (J), data are presented as the mean ± SEM; n.s., not significant; *P < 0.05, **P < 0.01, and ***P < 0.001, *t* test.

Emerging evidence has linked chronic stress with increased cancer risk (*4*, *5*). To investigate the impact of chronic stress on hepatocarcinogenesis within the native liver microenvironment, we used an orthotopic murine liver cancer model. Liver cancer was induced in 8-week-old C57BL/6J mice via hydrodynamic tail vein injection (HTVi) of plasmids encoding the sleeping beauty transposase (SBT) and transposons carrying human oncogenes *Akt* and *Nras* (termed Akt;Nras;SBT hereafter) (Fig. 2, A to C) (*27*). In this well-characterized system, these oncogenes are specifically overexpressed in hepatocytes recapitulating de novo hepatocarcinogenesis with hepatocytes as the cell of origin. This orthotopic model faithfully reproduces both the histopathological features of human liver cancer and the intricate interactions between tumor cells and the microenvironment.

C57BL/6J mice were subjected to 2 weeks of CRS followed by HTVi of Akt;Nras;SBT to induce liver cancer (Fig. 2D). Mice that received HTVi of Akt;Nras;SBT without prior CRS served as controls. While both groups maintained comparable body weights at baseline and endpoint, CRS induced pronounced weight loss (Fig. 2E), confirming effective chronic stress modeling. Mice in both groups developed macroscopic liver tumors (Fig. 2F). Histopathological analysis of tumor burden revealed that a tumor–to–liver area ratio, tumor number, and tumor size were markedly increased in CRS-exposed mice when compared to controls (Fig. 2, G and H). Immunohistochemical analysis demonstrated a significantly higher proportion of Ki67^+^ proliferating cells in CRS-exposed mouse livers (Fig. 2, I and J). Furthermore, serum levels of ALT, AST, alkaline phosphatase (ALP), and total bile acid (TBA) were substantially elevated in CRS-exposed liver cancer mice (Fig. 2K), indicating aggravated liver injury. These data establish that chronic stress exacerbates tumor progression in Akt;Nras-driven liver cancer.

### Chronic stress accelerates hepatocarcinogenesis in ∆N90-β-catenin;c-Met–driven HCC

**Fig. 3.**
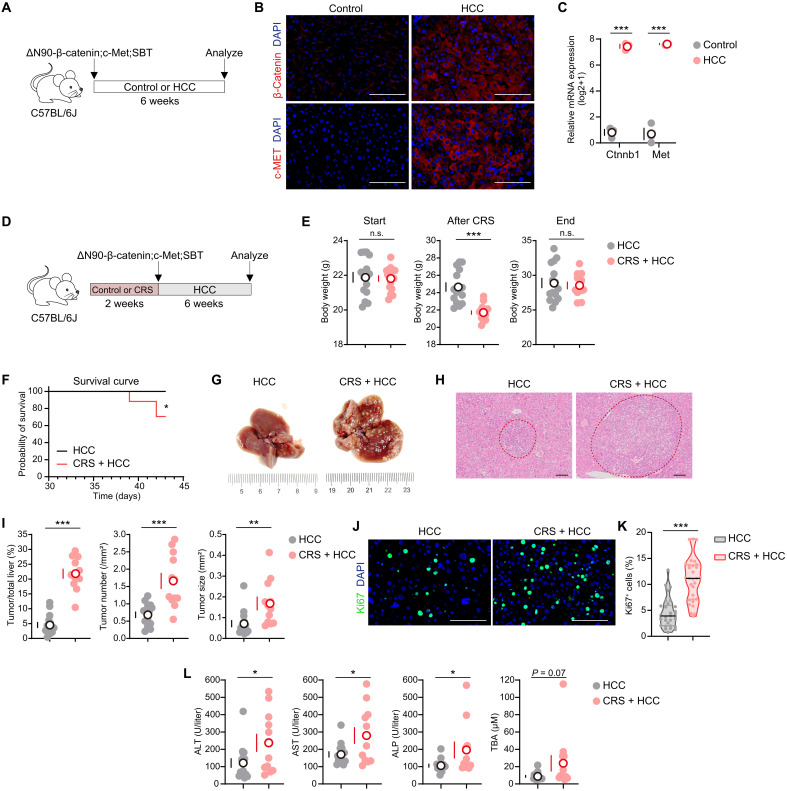
Chronic stress accelerates hepatocarcinogenesis in ∆N90-β-catenin;c-Met-driven HCC. (**A**) Schematic view of HCC modeling. Eight-week-old mice were induced HCC by HTVi of ∆N90-β-catenin;c-Met;SBT. Mice left undisturbed served as controls. All mice were euthanized 6 weeks later to quantify the expression of β-catenin and c-Met (B and C). (**B**) Representative immunofluorescence images. (**C**) qPCR measured the mRNA levels of *Ctnnb1* (which encodes β-catenin) and *Met* (*n* = 3). Data were presented as log_2_(expression+1). (**D**) Schematic view of CRS treatment and HCC modeling. Eight-week-old mice were subjected to CRS or left undisturbed. After 2 weeks, all mice were HTVi of ∆N90-β-catenin;c-Met;SBT to induce HCC (HCC, *n* = 15; CRS + HCC, *n* = 17). All mice were euthanized 6 weeks later to quantify the tumor formation [(E) to (L); HCC, *n* = 15; CRS + HCC, *n* = 12]. (**E**) Body weights at the start of the experiment, after 2-week restraint stress, and at the end of the experiment. (**F**) Survival analysis of mice in both groups. *P < 0.05, log-rank test. (**G**) Representative liver images. (**H**) Representative H&E staining images of liver sections. Tumors were circled by red lines. (**I**) The ratios of tumor/total liver area, tumor number per square millimeter, and tumor size. (**J**) Immunofluorescent staining of Ki67. (**K**) The ratio of Ki67^+^ cells in representative samples (*n* = 6). The median was indicated. (**L**) Serum levels of ALT, AST, ALP, and TBA. Scale bars, 100 μm. Except for (K), data are presented as the mean ± SEM; *P < 0.05, **P < 0.01, and ***P < 0.001, *t* test.

To further validate our findings, we used an additional oncogene-driven HCC model through HTVi of ΔN90-β-catenin;c-Met;SBT (Fig. 3, A to C) (*28*, *29*). Overexpression of β-catenin and c-Met in hepatocytes gave rise to histologically confirmed HCC within 6 to 8 weeks.

ΔN90-β-catenin;c-Met;SBT plasmids were hydrodynamically injected into C57BL/6J mice that were either subjected to prior CRS or served as unstressed controls (Fig. 3D). Analogously, while both groups maintained comparable body weights at the start and the final endpoint of the experiment, CRS-exposed mice exhibited significant poststress weight loss (Fig. 3E). Notably, CRS-exposed mice showed premature mortality (Fig. 3F), suggesting accelerated disease progression. Both groups developed macroscopic liver tumors (Fig. 3G). Histopathological analysis revealed increased tumor burden in CRS-exposed mice, including a markedly higher tumor–to–liver area ratio, tumor number, and tumor size (Fig. 3, H and I). Proliferation was significantly enhanced in CRS-exposed mouse tumors, as evidenced by the elevated ratio of Ki67^+^ cells (Fig. 3, J and K). CRS-exposed mice also displayed higher ALT, AST, and ALP levels (Fig. 3L), indicating exacerbated hepatic dysfunction. These results collectively demonstrate that chronic stress accelerates liver cancer progression across different oncogenic drivers.

To model the effects of chronic stress on established tumors, we initiated a chronic stress regimen after tumor induction. Given that tumor development requires 4 to 8 weeks, we used a modified chronic unpredictable stress paradigm (*25*, *30*). This protocol extends beyond the conventional 2-week restraint period and incorporates variable daily stressors to prevent habituation, thereby better modeling sustained psychological stress after tumor establishment. Mice were subjected to this stress regimen beginning on day 3 after HTVi of plasmids. Mice receiving HTVi of the plasmids alone served as the controls. Consistently, stress-exposed mice developed markedly increased tumor burden in both *Akt*;*Nras*-driven and ∆N90-β-catenin;c-Met–driven liver cancer (fig. S4, A to H). Collectively, these findings confirm that chronic stress exacerbates tumor progression irrespective of whether chronic stress begins before or after tumor induction.

### Adrenergic signaling is involved in liver cancer progression

**Fig. 4.**
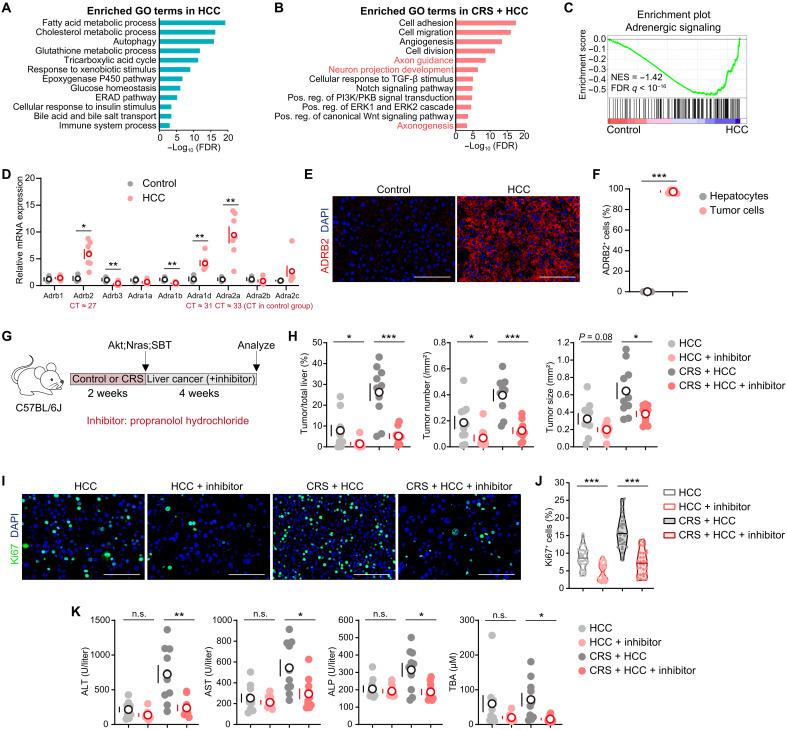
Adrenergic signaling is involved in Akt;Nras-driven liver cancer progression. (**A** and **B**) Enriched GO terms in HCC tissues (A) versus CRS + HCC tissues (B) in Akt;Nras;SBT-induced tumor model. (**C**) The GSEA analysis of the gene set of adrenergic signaling in Akt;Nras;SBT liver tissues versus control livers. (**D**) qPCR measured the mRNA levels of nine AR genes (control, *n* = 3; HCC, *n* = 7). (**E**) Representative immunofluorescence images of ADRB2. (**F**) The ratio of ADRB2^+^ cells (*n* = 5). (**G**) Schematic view of CRS treatment, tumor modeling, and propranolol treatment. Eight-week-old mice were subjected to CRS or left undisturbed. After 2 weeks, all mice were HTVi of Akt;Nras;SBT. Mice were then treated with propranolol or a vehicle control. All mice were euthanized 4 weeks later to quantify the tumor formation (H to K; *n* = 10). (**H**) The ratios of tumor/total liver area, tumor number per square millimeter, and tumor size. (**I**) Representative immunofluorescence images of Ki67. (**J**) The ratio of Ki67^+^ cells in representative samples (*n* = 6). The median was indicated. (**K**) Serum levels of ALT, AST, ALP, and TBA. Scale bars, 100 μm. Except for (J), data are presented as the mean ± SEM; *P < 0.05, **P < 0.01, and ***P < 0.001, *t* test. PI3K/PKB, phosphoinositide 3-kinase/protein kinase B; CT, cycle threshold; ERAD, endoplasmic reticulum-associated degradation.

To decipher the mechanisms underlying stress-accelerated liver cancer progression, we performed comparative transcriptomic analysis of CRS + HCC versus HCC livers in both Akt;Nras;SBT and ∆N90-β-catenin;c-Met;SBT models. Extrinsic driver signals are expected to be inferred based on the activated signaling pathways in CRS-exposed liver cancer samples. RNA-seq revealed distinct transcriptional profiles between CRS + HCC and HCC livers (fig. S5, A and B). Functional enrichment analysis of DEGs revealed that liver cancer samples were enriched in metabolic pathways (Fig. 4A), whereas CRS + HCC samples were enriched in protumorigenic pathways (Fig. 4B and fig. S5C). Neural signalings, such as neuron projection development, axon guidance, axonogenesis, and regulation of norepinephrine uptake, were enriched in CRS + HCC samples (Fig. 4B and fig. S5C). The liver is densely innervated by sympathetic nerves (*18*). Immunofluorescence confirmed increased density of tyrosine hydroxylase-positive (TH^+^) sympathetic nerve fibers in CRS-exposed livers (fig. S5D). During stress, sympathetic nervous system (SNS) activation triggers a fight-or-flight response, primarily through catecholamine release (*1*, *4*). Gene set enrichment analysis (GSEA) demonstrated significant enrichment of adrenergic signaling in liver cancer samples versus control healthy livers (Fig. 4C, fig. S5E, and table S1). These data raise the possibility that chronic stress accelerates liver cancer progression via SNS-derived catecholamine release and subsequent adrenergic signaling activation.

Adrenergic signaling is activated through cell surface ARs, including α-AR (ADRA1A, ADRA1B, ADRA1D, ADRA2A, ADRA2B, and ADRA2C) and β-AR (ADRB1, ADRB2, and ADRB3). Systematic expression analysis identified *Adrb2* (β2-AR) as the sole receptor gene both significantly up-regulated and highly expressed in liver tumors (Fig. 4D and fig. S5F). Immunofluorescence confirmed pronounced ADRB2 expression in liver cancer cells (Fig. 4, E and F, and fig. S5, G and H). Notably, pharmacological blockade of ADRB2 with propranolol markedly reduced tumor burden (Fig. 4, G and H). Consistent with this, the ratio of Ki67^+^ proliferating cells was significantly lower in propranolol-treated tumors (Fig. 4, I and J). Moreover, ALT, AST, ALP, and TBA levels were substantially reduced in propranolol-treated mice under chronic stress (Fig. 4K), indicating improved liver function. Crucially, however, the magnitude of reduction was more pronounced in CRS-exposed mice, consistent with our model that chronic stress exacerbates tumor progression by enhancing ADRB2 signaling, thereby creating a dependency that is particularly susceptible to β-blockade. In tumors exposed to stress after induction, we observed a marked enrichment of pathways including neuron projection development, axon guidance, and adrenergic signaling (fig. S5, I to K). Even when chronic stress was maintained after tumor induction, propranolol also markedly reduced tumor burden (fig. S5, L and M). These findings demonstrate that adrenergic signaling is involved in chronic stress–accelerated liver cancer progression.

### ADRB2 contributes to liver cancer progression

Immunofluorescence revealed prominent ADRB2 expression in tumor cells (Fig. 4, E and F, and fig. S5, G and H). However, previous studies have emphasized immune suppression in stress-induced tumor progression (*20*, *21*, *31*). To delineate the cellular mediators of chronic stress in liver cancer, we systematically mapped ADRB2 expression across the tumor microenvironment. snRNA-seq of liver cancer samples revealed detectable *Adrb2* transcripts in malignant cells and immune populations, including Kupffer cells, NKT cells, and B cells (fig. S6, A to P), albeit at low levels, due to limited transcript coverage in snRNA-seq datasets. Flow cytometry of disaggregated tumors provided higher-resolution protein-level data, confirming substantial ADRB2 expression on diverse immune populations, including monocytes, macrophages, dendritic cells, NK cells, T cells, and B cells (fig. S7, A to E). Notably, both CD4^+^ T cells and CD8^+^ T cells exhibited elevated ADRB2 levels in CRS-exposed liver tumors versus control liver tumors (fig. S7, D and E). This expression profiling establishes ADRB2 as a potential signaling node in both malignant and immune cells within the stressed liver tumor microenvironment.

**Fig. 5.**
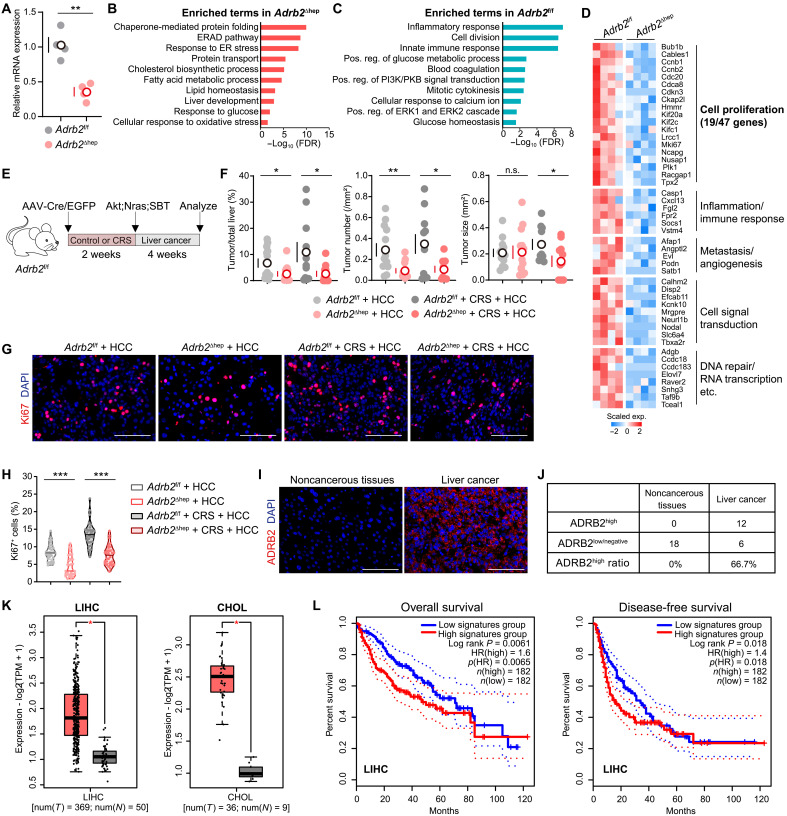
Hepatic ADRB2 contributes to liver cancer progression. (**A**) qPCR measured the mRNA levels of *Adrb2* in *Adrb2*^f/f^ and *Adrb2*^∆li^ hepatocytes (*n* = 4). (**B** and **C**) Enriched GO and KEGG terms in *Adrb2*^∆li^ hepatocytes (B) versus *Adrb2*^f/f^ hepatocytes (C). (**D**) Heatmap showed top down-regulated genes in *Adrb2*^∆li^ hepatocytes versus *Adrb2*^f/f^ hepatocytes. (**E**) Schematic view of *Adrb2* deletion, CRS treatment, and tumor modeling. *Adrb2*^f/f^ mice were divided into four groups: AAV-EGFP without CRS, AAV-Cre without CRS, AAV-EGFP followed by CRS, or AAV-Cre followed by CRS. After 2 weeks, all mice were HTVi of Akt;Nras;SBT. All mice were euthanized 4 weeks later to quantify the tumor formation [(F) to (H); *n* = 13, 13, 11, and 11, respectively]. (**F**) The ratios of tumor/total liver area, tumor number per square millimeter, and tumor size. (**G**) Representative immunofluorescence images of Ki67. (**H**) The ratio of Ki67^+^ cells in representative samples (*n* = 6). The median was indicated. (**I**) Immunofluorescent staining of ADRB2 in human liver cancer and adjacent nontumor tissues. (**J**) The ratio of ADRB2^high^ samples were quantified. (**K**) The expression levels of ADRB2 signaling genes in TCGA normal tissues (gray) and tumor tissues (red). LIHC, liver hepatocellular carcinoma; CHOL, intrahepatic cholangiocarcinoma. (**L**) On the basis of the overall expression of ADRB2 signaling genes in tumor tissues, patients were classified into the high signature group (top 50%) and the low signature group (bottom 50%). Kaplan-Meier analysis showed the overall survival (left) and disease-free survival (right) of patients with HCC in both groups. Scale bars, 100 μm. Except for (H), data are presented as the mean ± SEM; *P < 0.05, **P < 0.01, and ***P < 0.001, *t* test. ER, endoplasmic reticulum; HR, hazard ratio; TPM, transcripts per million.

While chronic stress is thought to promote cancer largely through immune suppression, its direct, tumor cell–intrinsic effects remain poorly defined. To functionally dissect the contribution of hepatic ADRB2 in liver cancer progression, we specifically deleted *Adrb2* in hepatocytes via delivering adeno-associated viruses (AAVs) 2/8-carried Cre recombinase (AAV-Cre) into *Adrb2*^f/f^ mice (Fig. 5A). We performed RNA-seq of ADRB2-deficient hepatocytes (*Adrb2*^∆hep^) and control hepatocytes (*Adrb2*^f/f^). Transcriptional profiles diverged between *Adrb2*^f/f^ and *Adrb2*^∆hep^ hepatocytes (fig. S7F). Pathway analysis revealed that *Adrb2*^∆hep^ hepatocytes were enriched in gene sets including protein folding, protein transport, and fatty acid metabolism (Fig. 5B), whereas *Adrb2*^f/f^ hepatocytes were enriched in gene sets including cell division, immune response, and glucose metabolism (Fig. 5C). Notably, 40.4% (19 of 47) of top down-regulated genes in *Adrb2*^∆hep^ hepatocytes were proliferation associated (Fig. 5D and table S1), implicating that ADRB2-mediated mitogenic signaling might contribute to liver cancer progression. Notably, *Adrb2*^∆hep^ mice exhibited a marked reduction in tumor burden (Fig. 5, E and F), accompanied by decreased Ki67^+^ proliferating cells (Fig. 5, G and H), and reduced ALP and TBA levels under chronic stress (fig. S7G). These data indicate that hepatic ADRB2 contributes to liver cancer progression.

**Fig. 6.**
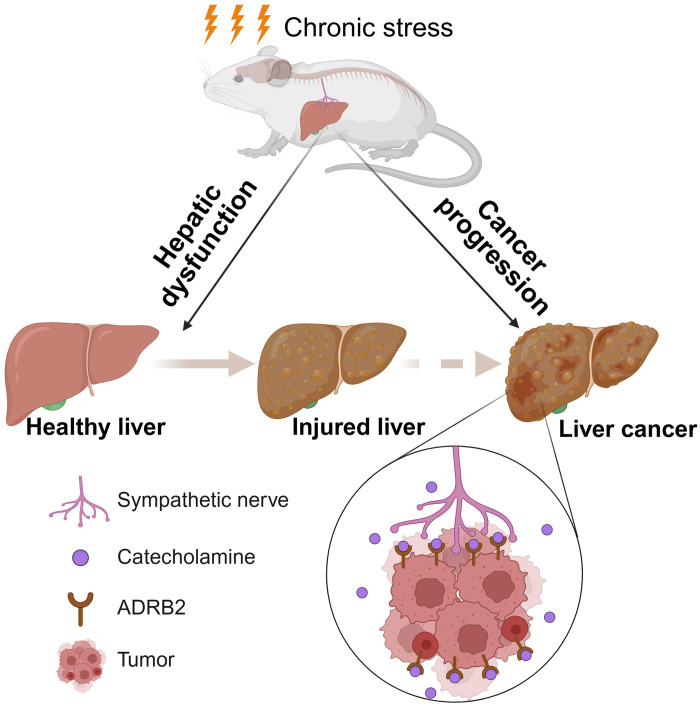
Summary of chronic stress–induced hepatic dysfunction and cancer progression. Chronic stress impairs hepatic homeostasis and exacerbates liver cancer progression through ADRB2 signaling. Created in BioRender. Li, L. (2026) https://BioRender.com/mn6v5t7.

To establish the clinical relevance of our findings, we interrogated ADRB2 protein expression in human liver cancer specimens. Immunofluorescence analysis revealed high ADRB2 expression in 66.7% (12 of 18) of tumors, whereas all matched noncancerous tissues were ADRB2 low or negative (Fig. 5, I and J), indicating specific up-regulation of ADRB2 in the malignant state. Elevated ADRB2 expression was associated with poor clinical outcomes in patients with HCC (*23*). To further elucidate the correlation between ADRB2 signaling and the clinical outcomes of patients with cancer, we analyzed The Cancer Genome Atlas (TCGA) datasets. Compared to TCGA normal livers, the overall expression of ADRB2 signaling genes (table S1) was significantly up-regulated in both liver hepatocellular carcinoma (LIHC) and intrahepatic cholangiocarcinoma (CHOL; Fig. 5K). High ADRB2 signaling correlated with reduced overall survival and disease-free survival in patients with LIHC (Fig. 5L). This pattern also extended to other tumor types including adrenocortical carcinoma (fig. S8A), pancreatic adenocarcinoma (fig. S8B), and brain lower-grade glioma (fig. S8C). In summary, our study demonstrates that chronic stress disrupts hepatic homeostasis and accelerates liver cancer progression via ADRB2 signaling, suggesting that targeting ADRB2 signaling may be applied to develop antitumor therapies (Fig. 6).

## DISCUSSION

There are intricate anatomical connections and functional communications between the central nervous system and peripheral organs (*32*). The past few years have been a surge in exploring the impact of stress on peripheral tissue function. Notably, our collaborative partners previously demonstrated that an appropriate strength and form of stress enhance adaptive immunity in the spleen through a distinct brain-spleen neural connection (*33*). Although chronic stress has been linked to steatohepatitis (*34*), a comprehensive pathophysiological profile is lacking. In the present study, by using the canonical chronic stress paradigm in conjunction with single-cell resolution transcriptome profiling, blood biochemistry, and histological analyses, we systematically delineated chronic stress–induced hepatic dysfunction. Our data reveal that chronic stress triggers hyperglycemia primarily through dysregulated hepatic gluconeogenesis, evidenced by the specific up-regulation of the key gene *Pck1*, rather than through systemic insulin deficiency. This metabolic dysregulation likely stems from the sustained activation of neuroendocrine pathways, including the hypothalamic-pituitary-adrenal (HPA) axis, sympathetic-adrenal-medullary (SAM) axis, or brain-liver projections, akin to that implicated in acute stress–induced hyperglycemia (*35*, *36*). Beyond metabolism, chronic stress also perturbs hepatic immunity. We observed a reduction in the proportion of Kupffer cells, the liver-resident macrophages essential for immune surveillance and liver repair (*37*). Following chronic stress, the disrupted immune system may produce excess proinflammatory cytokines, amplifying inflammation and oxidative stress within the liver, thus culminating in liver cell damage (*38*, *39*). Our findings support the notion that psychological states play an important role in tissue homeostasis and overall health. It would be intriguing to characterize the mechanisms underlying chronic stress–induced liver injury in the future.

Clinical and epidemiological evidence suggests that chronic stress fosters tumor development and increases mortality in patients with cancer (*4*, *5*, *40*, *41*). However, a critical gap persists in understanding its direct role in tumor progression, as most mechanistic studies have relied on subcutaneous xenograft models and exogenous catecholamine administration (*20*–*23*). These approaches are limited because xenografts lack a native tissue microenvironment, and acute catecholamine delivery fails to recapitulate the spatiotemporal dynamics of physiological stress. To overcome these limitations, we used two orthotopic murine liver cancer models. These systems enable us to study oncogene-driven tumorigenesis within the native liver microenvironment under physiologically relevant chronic stress paradigms, including CRS and chronic unpredictable stress. Our findings provide compelling evidence that chronic stress potently promotes orthotopic liver cancer progression in vivo. These findings align with prior observations that social isolation stress reduces the survival time of mice with liver cancer (*42*).

The emerging field of cancer neuroscience suggests that the nervous system actively modulates tumor progression, and chronic stress may act through this interface (*43*, *44*). We observed that CRS-exposed mice exhibited elevated neuronal activation across key mood regulatory brain regions, suggesting potential central regulations for stress to influence peripheral tumors. Chronic stress activates both the HPA axis and the SNS, which can affect cancer by releasing stress factors, including norepinephrine, epinephrine, and glucocorticoids (*4*, *45*). While glucocorticoids exhibit context-dependent pro- or antitumor effects via pleiotropic regulation of cell growth, metabolism, and immunity (*46*–*48*), catecholamines such as norepinephrine and epinephrine consistently emerge as potent tumor promoters (*9*, *11*, *49*–*51*). The liver, receiving dense sympathetic innervation, is a prime target for neural regulation (*18*, *52*). Clinically, elevated sympathetic fiber density in HCC specimens correlates with poor outcomes (*53*), and down-regulation of the catecholamine-degrading enzyme monoamine oxidase A is also linked to poor outcomes in patients with HCC (*54*). In addition to direct neuronal input, the liver, as a highly perfused organ, is also exposed to circulating catecholamines released via the SAM axis (*1*, *4*), providing an alternative route for ADRB2 activation. Here, we propose a mechanistic axis wherein stress-activated catecholamine release stimulates ADRB2 signaling, thereby accelerating HCC progression.

General consensus attributes stress-induced tumor progression primarily to immune suppression (*20*, *21*, *31*). Our data identify a previously underappreciated direct effect on cancer cells. The broad expression of ADRB2 on both tumor cells and immune cells suggests a complex cellular network within the tumor microenvironment. Under chronic stress, elevated catecholamine release simultaneously activates ADRB2 signaling in both tumor cells and immune cells (*55*). In tumor cells, this signaling directly drives cell proliferation via cyclic adenosine monophosphate (cAMP)-protein kinase A (PKA)–mediated activation of extracellular signal–regulated kinase (ERK) and AKT (also known as PKB) pathways (*55*). In parallel, ADRB2 activation fosters exhaustion in T cells, thereby establishing an immunosuppressive niche for tumor growth (*31*, *56*, *57*). This allows stress signals to co-opt both the malignant cells and the niche to accelerate cancer progression. Notably, targeting β-AR has emerged as a potential strategy to reduce HCC risk (*58*, *59*). β-AR blockers have demonstrated therapeutic efficacy in patients with pancreatic cancer (*60*) and patients with breast cancer (*61*). Our work provides a mechanistic rationale for repurposing these drugs, particularly in patients exhibiting high tumoral ADRB2 expression or reporting high stress levels, to improve therapeutic outcomes.

Despite the interesting findings, the study is not without limitations. In this study, we demonstrate that chronic stress exacerbates liver injury and fuels cancer growth by activating ADRB2 signaling. However, we did not identify the precise neuroanatomical origins within the central nervous system. The specific brain regions and the neural circuits that modulate hepatic ADRB2 activation remain to be elucidated. Future studies using neural tracing, organ-specific denervation, and cell type–specific modulation techniques will be essential to illustrate the central-peripheral circuits governing this brain-liver axis. Despite these limitations, our findings establish chronic stress as a risk factor for hepatic disorders and position ADRB2 blockade as a promising therapeutic strategy for liver malignancies.

## MATERIALS AND METHODS

### Mice

C57BL/6J mice were purchased from Shanghai Jihui Laboratory Animal Care Co. Ltd. *Adrb2*^f/f^ mice were provided by J. Yang (Peking University, China). All mouse experiments were approved by the Animal Care and Use Committee of Shanghai General Hospital (approval number: 2024AW015) and ShanghaiTech University (approval number: 20250507002) and performed in accordance with the committee’s guidelines.

None of the mice used in the study had been subjected to any prior procedures. Mice were housed in a temperature- and light-controlled (12-hour light/dark cycle with light from 7:00 to 19:00) specific pathogen–free animal facility in individually ventilated cages with companion mice, except for specified isolation protocol. Mice used in the study were 8 to 16 weeks of age. To minimize selection bias, mice were randomized into different groups before any experimental procedures. Male mice were used for most experiments, unless otherwise specified. Mouse samples were routinely collected during the light phase from Zeitgeber Time (ZT) 6 to ZT 12. The blood samples presented in fig. S3 (A to D), however, were specifically collected from ZT 8 to ZT 10. For β-AR antagonist studies, propranolol hydrochloride (MedChemExpress, HY-B0573), at a daily dosage of 40 mg/kg, or a water vehicle was administered to mice via their drinking water. For AAV virus infection, 3 × 10^11^ genomic particles of AAV2/8-TBG-Cre (BrainVTA, PT-0576) or AAV2/8-TBG-EGFP (BrainVTA, PT-0824) were reconstituted in 200 ml of phosphate-buffered saline (PBS) and injected intravenously through tail veil injection with BD Ultra-Fine Insulin Syringes (*37*).

### Human liver cancer tissues

Human liver cancer tissue microarrays (HLiv-mix036PG-01) were commercially obtained from Shanghai Outdo Biotech Co. Ltd. The utilization of these deidentified tissue samples was conducted in compliance with the ethical standards set forth by the Ethics Committee of Shanghai General Hospital (approval number: 2025KS765) and the Ethics Committee of the Shanghai Outdo Biotech (approval number: YB M-05-01), under which the original collection of tissues was performed with patient informed consent.

### Orthotopic liver cancer models

Liver cancer was induced via HTVi as previously described (*28*). Briefly, 1.6 μg of plasmids encoding the SBT (pT3-CMV-SBT), 10 μg of plasmids encoding transposons carrying human ΔN90-β-catenin (pT3-EF1a-ΔN90-β-catenin), and 10 μg of plasmids encoding transposons carrying human c-Met (pT3-EF1a-c-Met) were diluted in 2.5 ml of sterile 0.9% NaCl solution. The plasmid solution was injected into the lateral tail veins of mice at a volume equivalent to 10% of the body weight (e.g., 2.5 ml for a 25-g mouse) in 5 to 7 s. Plasmids can be efficiently delivered into hepatocytes through the vena cava and central vein via relatively high-pressure tail vein injection. Ectopic expression of ΔN90-β-catenin and c-Met in mouse hepatocytes efficiently induced HCC within 6 to 8 weeks (*29*). Similarly, ectopic expression of *Nras* (pT3-Caggs-Nras) and *Akt* (pT3-EF1a-Akt) in mouse hepatocytes efficiently induced liver cancer within 4 to 5 weeks (*27*).

### Chronic stress paradigms

To analyze the effects of chronic stress on hepatic homeostasis, mice were subjected to CRS as previously described (*24*, *25*). Briefly, mice were individually placed in 50-ml tubes for 3 hours per day over 2 weeks (designated as the CRS group). The restraint protocol prevented mice from moving freely but did not press them. Age- and sex-matched mice without restraint were used as controls (designated as the control group). To analyze the effects of chronic stress on liver cancer progression, 8-week-old C57BL/6J mice were subjected to 2 weeks of CRS and then HTVi of Akt;Nras;SBT or ΔN90-β-catenin;c-Met;SBT to induce liver cancer (designated as the CRS + HCC group). Mice with HTVi of Akt;Nras;SBT or ΔN90-β-catenin;c-Met;SBT, but without stress treatment, served as controls (designated as the HCC group).

The chronic stress regimen was extended after tumor induction to better mimic its effect on cancer progression. Mice were subjected to a panel of stressors from day 3 after HTVi of plasmids until the end of the experiment (designated as the HCC + stress group). The chronic stress procedure (*25*, *30*) with certain modifications followed a randomized weekly schedule of commonly used stressors to prevent habituation: (i) social isolation; (ii) tube restraint (3 hours); (iii) water deprivation (18 hours); (iv) tail clipping (10 min); (v) overnight illumination (12 hours); (vi) soiled cages (200 ml of water in the sawdust bedding, 24 hours); and (vii) foot shock (1.75 mA, 40 min). Mice that received HTVi of the plasmids alone served as controls (designated as the HCC group).

### OGTT and ITT

Mice were fasted for 16 hours for oral glucose tolerance test (OGTT) or fasted for 6 hours for insulin tolerance test (ITT) (*62*). Blood glucose measurements were performed during the light phase between ZT 8 and ZT 10. Blood glucose levels were measured from the tail vein using a handheld glucometer (Roche). For the OGTT, the fasted mice received an oral gavage of glucose (2 g per kg of body weight, delivered as a 20% solution in double-distilled water. Blood glucose levels were monitored at 0, 15, 30, 60, 90, and 120 min postadministration. For the ITT, the fasted mice were intraperitoneally injected with recombinant human insulin (Yeasen, 40112ES25) at a dose of 0.75 U per kg of body weight. Blood glucose levels were monitored at 0, 15, 30, 45, 60, 90, and 120 min postinjection.

### Blood biochemical analysis

Blood samples were routinely collected from ZT 6 to ZT 12 and stored at 4°C for 1 hour. Serum was then isolated by centrifugation at 7500*g* for 10 min (4°C) and stored at −80°C for subsequent use. Serum levels of ALT, AST, ALP, TBA, and glucose (GLU) (in Fig. 1F) were measured using a fully automated biochemical analyzer (Biobase, BK-280). Insulin levels were measured using enzyme-linked immunosorbent assay kits (Jonlnbio, JL11459) according to the manufacturer’s protocol.

Blood was collected into 1.5-ml tubes containing anticoagulants. Plasma was immediately obtained by centrifugation at 2000*g* for 15 min at room temperature. APTT was measured using an automated coagulation monitoring device (Teco, MC-4000) with a maximum recorded value of 96 s as previously described (*37*).

### Histology and immunofluorescent staining

For frozen sections, livers were fixed in 4% paraformaldehyde (PFA; 4°C) for 1 hour, and brains were fixed in 4% PFA (4°C) overnight, followed by dehydration in 30% sucrose (4°C) overnight before freezing in OCT (optimal cutting temperature compound) tissue blocks. Oil Red O staining was performed on frozen liver sections (8 μm). For immunofluorescent staining, frozen sections (liver, 5 μm; brain, 40 μm) were washed in PBST (0.1% Triton X-100 in PBS) for 15 min × 2, blocked in 10% bovine serum albumin (BSA) for 1 hour at room temperature, and incubated with primary antibody at 4°C overnight. Primary antibodies included c-Fos (1:2000; Cell Signaling Technology, 2250) and tyrosine hydroxylase [(TH); 1:1000; Millipore, AB152). Following primary antibody incubation, sections were incubated with fluorophore-conjugated secondary antibodies (Jackson ImmunoResearch, 711-545-152), counterstained with DAPI (4′,6-diamidino-2-phenylindole) for nuclei visualization, and mounted with antifade mounting medium.

For paraffin sections, liver samples were fixed in 4% PFA (4°C) overnight and embedded in paraffin blocks the next day. Hematoxylin and eosin (H&E) and immunofluorescent staining were performed on these paraffin sections as previously described (*37*). For immunofluorescent staining, 4-μm paraffin sections were subjected to antigen retrieval. Subsequently, sections were washed with TBST (0.1% Tween 20 in tris-buffered saline) for 2 min, blocked with antibody diluent for 1 hour at room temperature, and incubated with primary antibodies at 4°C overnight. Primary antibodies included c-MET (1:500; Cell Signaling Technology, 8198), β-catenin (1:200; Proteintech, 51067-2-AP), AKT (1:1000; Santa Cruz, sc-5298), RAS (1:500; Cell Signaling Technology, 67648), ADRB2 (1:500; Abcam, ab182136), Ki67 (1:1000; Cell Signaling Technology, 9129), CLEC4F (1:200; R&D Systems, AF2784), F4/80 (1:1000; Cell Signaling Technology, 70076), and VSIG4 (1:500; Abcam, ab252933). Primary antibody signals were detected using a three-color fluorescence kit (Recordbio, RC0086-23) following the manufacturer’s instructions. Sections were stained with DAPI and mounted with antifade mounting medium. Images were captured using a Leica Mateo FL microscope, a Zeiss Z2 microscope, or an Olympus VS120 microscope. Multichannel images were merged and processed using ImageJ software.

### Flow cytometry

Tumor tissues were harvested from Akt;Nras;SBT mice and CRS-exposed Akt;Nras;SBT mice. Visible liver tumors were carefully dissected and minced into small pieces using sterile surgical scissors. The tissue fragments were then digested in Dulbecco’s modified Eagle’s medium supplemented with collagenase IV (1 mg/ml; Life Technologies, 17104019) and deoxyribonuclease I (0.2 mg/ml; Solarbio, D8071) for 30 min in a 37°C water bath. The digestion process was terminated with 5% fetal bovine serum. All liver cells were filtered through a 40-μm cell strainer for subsequent staining and flow cytometry analysis as previously described (*37*). Following Fc receptor blockade (anti-mouse CD16/CD32, Bio X Cell, BE0307), cells were stained for 30 min at 4°C with a cocktail of fluorophore-conjugated antibodies diluted in fluorescence-activated cell sorting buffer at 1:200. Antibodies included CD45.2-Brilliant Violet (BV) 510 (BioLegend, 109838), CD8a-BV650 (BioLegend, 100742), CD4–phycoerythrin (PE)/cyanine 7 (Cy7) (BioLegend, 100421), NK1.1-allophycocyanin (APC)/Cy7 (BioLegend, 108723), CD45R/B220-phycoerythrin (PE) (BioLegend, 103207), Ly6C-BV785 (BioLegend, 128041), Ly6G-BV711 (BioLegend, 127643), F4/80-BV421 (BioLegend, 123131), CD11b-BV605 (BioLegend, 101237), CD11c-APC (BioLegend, 117310), and MHC Class II-Peridinin Chlorophyll Protein (MHCII-PerCP)/Cy5.5 (BioLegend, 107625). Dead cells were excluded with Live/Dead Dye (Fixable Viability Stain 700, BD Bioscience, 564997). After surface marker staining, cells were fixed and permeabilized using the Fixation/Permeabilization Buffer (Thermo Fisher Scientific, 00-5223-00) according to the manufacturer’s protocol. Intracellular ADRB2 was labeled by incubation with a primary antibody (1:200; Thermo Fisher Scientific, MA5-32570), followed by a fluorophore-conjugated secondary antibody (donkey anti-rabbit immunoglobulin G–AF488, BioLegend, 406416). All samples were acquired on a BD LSR Fortessa flow cytometer, and data were analyzed using FlowJo software (v10).

### Magnetic-activated cell sorting

Mouse livers were digested by a modified two-step collagenase IV (Life Technologies, 17104019) perfusion method. Liver cells were filtered through a 70-μm cell strainer. Hepatocytes were purified by a series of low-speed gravity centrifugation (50*g* × 1 min × 3). NPCs, including endothelial cells, hepatic stellate cells, biliary epithelial cells, Kupffer cells, neutrophils, and intrahepatic lymphocytes, were purified by low-speed gravity centrifugation (50*g* × 1 min × 2) and medium-speed gravity centrifugation (300*g* × 5 min × 1) as previously described (*37*). NPCs were resuspended in sorting buffer (PBS with 0.5% BSA and 2 mM EDTA), filtered through a 40-μm cell strainer, and incubated with Fc block anti-mouse CD16/32 antibody (FineTest, FNab30106) for 15 min at 4°C. Cells were then labeled with PE-conjugated anti–TIM-4 (T cell immunoglobulin and mucin domain-containing protein 4) antibody (Miltenyi Biotec, 130-116-757) for 15 min at 4°C followed by anti-PE MicroBeads (Miltenyi Biotec, 130-048-801) for 15 min at 4°C. Magnetic separation was performed using magnetic-activated cell sorting (MACS) mass spectrometry columns according to the manufacturer’s protocol. The TIM-4–positive fraction (TIM-4^+^ cells) and unlabeled cell fraction (unlabeled NPCs) were collected. To characterize the sorted TIM-4^+^ cell population, RNA was isolated from TIM-4^+^ cells, unlabeled NPCs, and hepatocytes for qPCR analysis of canonical Kupffer cell markers *Adgre1*, *Clec4f*, *Vsig4*, and *Timd4* (*37*, *63*).

### RNA isolation, qPCR, and bulk RNA-seq

Mouse livers were perfused with 0.9% NaCl solution to obtain liver cells. Hepatocytes were isolated via a modified two-step collagenase IV (Life Technologies, 17104019) perfusion protocol. The resulting cell suspension was passed through a 70-μm cell strainer, and hepatocytes were further purified by a series of low-speed gravity centrifugation (50*g* × 1 min × 3) as previously described (*37*). Total RNA of purified hepatocytes or all liver cells was extracted using TRIzol reagent (Invitrogen, 15596018CN) following the manufacturer’s protocol.

RNA concentration was measured, and 1 μg of total RNA was reverse transcribed into cDNA using a commercial kit (Vazyme, R323-01). Quantitative real-time polymerase chain reaction (qPCR) was then performed on an ABI StepOnePlus system (Applied Biosystems) with SYBR Green master mix (Vazyme, Q312-02). Primers for qPCR are provided in table S2. Gene expression levels were normalized to the endogenous reference gene *Gapdh*.

For bulk RNA-seq, libraries were constructed from 1 μg of total RNA using the Illumina TruSeq RNA Sample Prep Kit. Paired-end sequencing (150-bp reads) was performed on an Illumina HiSeq 4000 sequencer by Annoroad Gene Technology Co. Ltd. (*37*).

### Single-nucleus RNA sequencing

For snRNA-seq, the 10× chromium pipeline was applied. CRS-exposed mice, control mice, Akt;Nras;SBT mice, CRS-exposed Akt;Nras;SBT mice, ΔN90-β-catenin;c-Met;SBT mice, and CRS-exposed ΔN90-β-catenin;c-Met;SBT mice were perfused with 0.9% NaCl solution to remove blood cells. Mouse livers were immediately snap-frozen in liquid nitrogen and stored at −80°C before use.

Nuclear isolation was performed on ice using nuclear lysis buffer followed by filtration, centrifugation, and washing. After nuclear isolation and quality control, single-nucleus libraries were prepared according to the manufacturer’s protocol of Chromium Next GEM Automated Chip G Single Cell Kit (10× Genomics). Briefly, six snRNA-seq libraries containing 7375 nuclei from control mouse livers, 12,566 nuclei from CRS mouse livers, 14,096 nuclei from Akt;Nras;SBT mouse livers, 13,175 nuclei from CRS-exposed Akt;Nras;SBT mouse livers, 6778 nuclei from ΔN90-β-catenin;c-Met;SBT mouse livers, and 8259 nuclei from CRS-exposed ΔN90-β-catenin;c-Met;SBT mouse livers were constructed and sequenced on an Illumina NovaSeq platform by Annoroad Gene Technology Co. Ltd.

### snRNA-seq data analysis

Cell Ranger software (v7.1.0, 10× Genomics) was applied to demultiplex the Illumina binary base call (BCL) output into FASTQ files. The Cell Ranger count was then applied to each FASTQ file to align reads to the reference genome and generate barcode and unique molecular identifier counts. We followed the Seurat (v5.3.0) (*64*) integrated analysis and comparative analysis workflows to do snRNA-seq analyses. For quality control and filtering out low-quality cells, only cells with nFeature_RNA between 200 and 10,000 and mitochondrial genes fewer than 5% were retained.

The datasets were integrated on the basis of “anchors” identified between datasets (nfeatures = 3000, normalization.method = “LogNormalize”) before performing linear dimensional reduction by principal components analysis (PCA). Batch effect correction was performed using Harmony (v1.2.3) (*65*) with max.iter.harmony = 20 and dims.us = 1:40. scDblFinder (v1.22.0) (*66*) was used to detect and remove potential doublets with parameters nfeatures = 3000 and default dbr. The UMAP (Uniform Manifold Approximation and Projection) method was used for visualization of unsupervised clustering. The top 25 PCs were included in a UMAP dimensionality reduction. Differential gene expression or marker genes was determined by the “findMarkers” function with the default Wilcoxon’s rank sum test either as one versus the rest or as a direct comparison with parameters min.pct = 0.25 and logfc.threshold = 0.25. Cell cluster identities were determined manually by using cell type–specific markers.

Following rigorous quality control and data filtration procedures, high-quality transcriptomic profiles from 6554 cells in control mice, 11,073 cells in CRS-exposed mice, 11,651 cells in Akt;Nras;SBT mice, 10,922 cells in CRS-exposed Akt;Nras;SBT mice, 5603 cells in ΔN90-β-catenin;c-Met;SBT mice, and 6678 cells in CRS-exposed ΔN90-β-catenin;c-Met;SBT mice were obtained for downstream analyses. Cell clusters were identified by graph-based clustering and visualized using UMAP. Major cell populations were identified using established cell type–specific marker genes, including hepatocytes (*Hnf4a*^+^, *Azgp1*^+^, *Nudt7*^+^, and *Ces1c*^+^), endothelial cells (*Stab2*^+^, *Ptprb*^+^, and *Tek*^+^), hepatic stellate cells (*Reln*^+^, *Colec10*^+^, *Lrat*^+^, and *Dcn*^+^), Kupffer cells (*Adgre1*^+^, *Clec4f*^+^, *Vsig4*^+^, and *Timd4*^+^), NKT cells (*Skap1*^+^, *Klrb1c*^+^, *Cd3e*^+^, and *Cd226*^+^), B cells (*Ebf1*^+^, *Pax5*^+^, *Cd19*^+^, and *Ighm*^+^), and cholangiocytes (*Pkhd1*^+^, *Spp1*^+^, *Thsd4*^+^, *Bicc1*^+^, *Hnf1b*^+^, and *Anxa4*^+^). In addition, liver cancer cells (*Prom1*^+^, *Afp*^+^, and *Gpc3*^+^), proliferating cells (*Mki67*^+^, *Ccnb2*^+^, and *Cdkn3*^+^), interferon response cells (*Ifit2*^+^, *Socs1*^+^, and *Isg15*^+^), and reactive cholangiocytes or liver cancer cells coexpressing both hepatocytes and cholangiocytes markers were identified.

### Bulk RNA-seq data analysis

The sequencing reads were mapped to the mm10 using STAR (v2.7.10a) (*67*). The RSEM (RNA-Seq by Expectation-Maximization, v1.3.1) (*68*) was used to estimate the number of reads mapped to each gene. The DESeq2 (v1.34.0) (*69*) R package was used to detect DEGs between two groups. DAVID (*70*) was used to perform enrichment analysis of DEGs. GSEA (v4.3.0) (*71*) was used to detect gene sets that exhibited significant differences between two groups. Heatmap and PCA plots were generated using R packages. The NAFLD genes, HCC progression signature genes, and adrenergic signaling genes are listed in table S1.

### TCGA data analysis

The study used publicly available data from TCGA project. TCGA data analysis was performed using the GEPIA2 website (http://gepia2.cancer-pku.cn/) (*72*). ADRB2 signaling genes are listed in table S1.

### Statistical analysis

All experimental data were presented as the mean ± SEM unless otherwise specified. “*n*” represented the number of animals and was indicated in the figures and figure legends. For quantification of Oil Red O–stained hepatocytes in liver sections, three random fields of each liver sample were quantified using ImageJ. For quantification of Ki67^+^ cells in liver sections, five random fields of each liver sample were quantified using ImageJ. Tumor/total liver area, tumor number, and tumor size in liver sections were quantified using QuPath (v0.5.0) (*73*). No statistical method was used to predetermine the sample size. Sample processing was not blinded. For statistical evaluation, an unpaired two-sided Student’s *t* test was performed using GraphPad Prism software (v8) unless otherwise stated and mentioned in the figure legends.
