# Supplementary material for: Chronic stress disrupts hepatic homeostasis and accelerates liver cancer progression through ADRB2 signaling
Source: Sci Adv. 2026 May 1;12(18):eaec0825. doi: 10.1126/sciadv.aec0825 (PMC13134585; doi:10.1126/sciadv.aec0825)
Supplement: Supplementary file 1 — Figs. S1 to S8 Legends for tables S1 and S2 Legend for data file S1 [file sciadv.aec0825_sm.pdf]

Science Advances
AAAS

Supplementary Materials for

Chronic stress disrupts hepatic homeostasis and accelerates liver cancer progression through ADRB2 signaling

Huimin Qin *et al.*

Corresponding author: Yue Lan, bluemooning@163.com; Ji Hu, huji@shanghaitech.edu.cn;
Lu Li, lu.li@sjtu.edu.cn

Sci. Adv. **12**, eaec0825 (2026)
DOI: 10.1126/sciadv.aec0825

The PDF file includes:

Figs. S1 to S8
Legends for tables S1 and S2
Legend for data file S1

Other Supplementary Material for this manuscript includes the following:

Tables S1 and S2
Data file S1

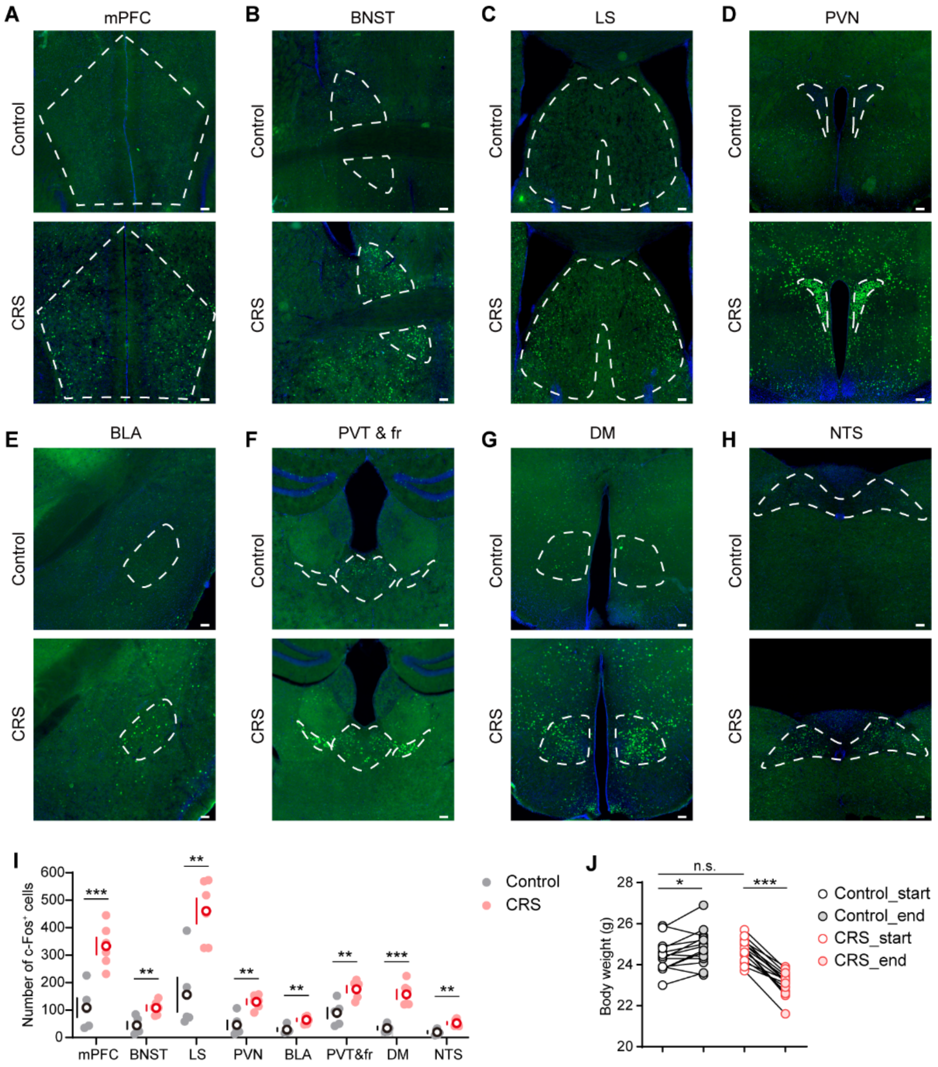

Fig. S1. Increased c-Fos-positive cells in various brain regions in CRS mice. (**A** to **H**) Representative immunofluorescence images of c-Fos in brain sections of control and CRS mice. A, mPFC, medial prefrontal cortex. B, BNST, bed nucleus of the stria terminalis. C, LS, lateral septum. D, PVN, paraventricular nucleus of the hypothalamus. E, BLA, basolateral amygdala. F, PVT&fr, paraventricular thalamus nucleus & fasciculus retroflexus. G, DM, dorsomedial hypothalamic nucleus. H, NTS, nucleus of the solitary tract. (**I**) The number of c-Fos^{+} cells in representative brain regions (Control, n = 5; CRS, n = 6). (**J**) Body weights of control mice (n = 14) and CRS-exposed mice (n = 15) at the start and end of experiments. Scale bars: 100 µm. Data are presented as the mean ± SEM; n.s., not significant; *$P < 0.05$, **$P < 0.01$, ***$P < 0.001$, *t*-test.

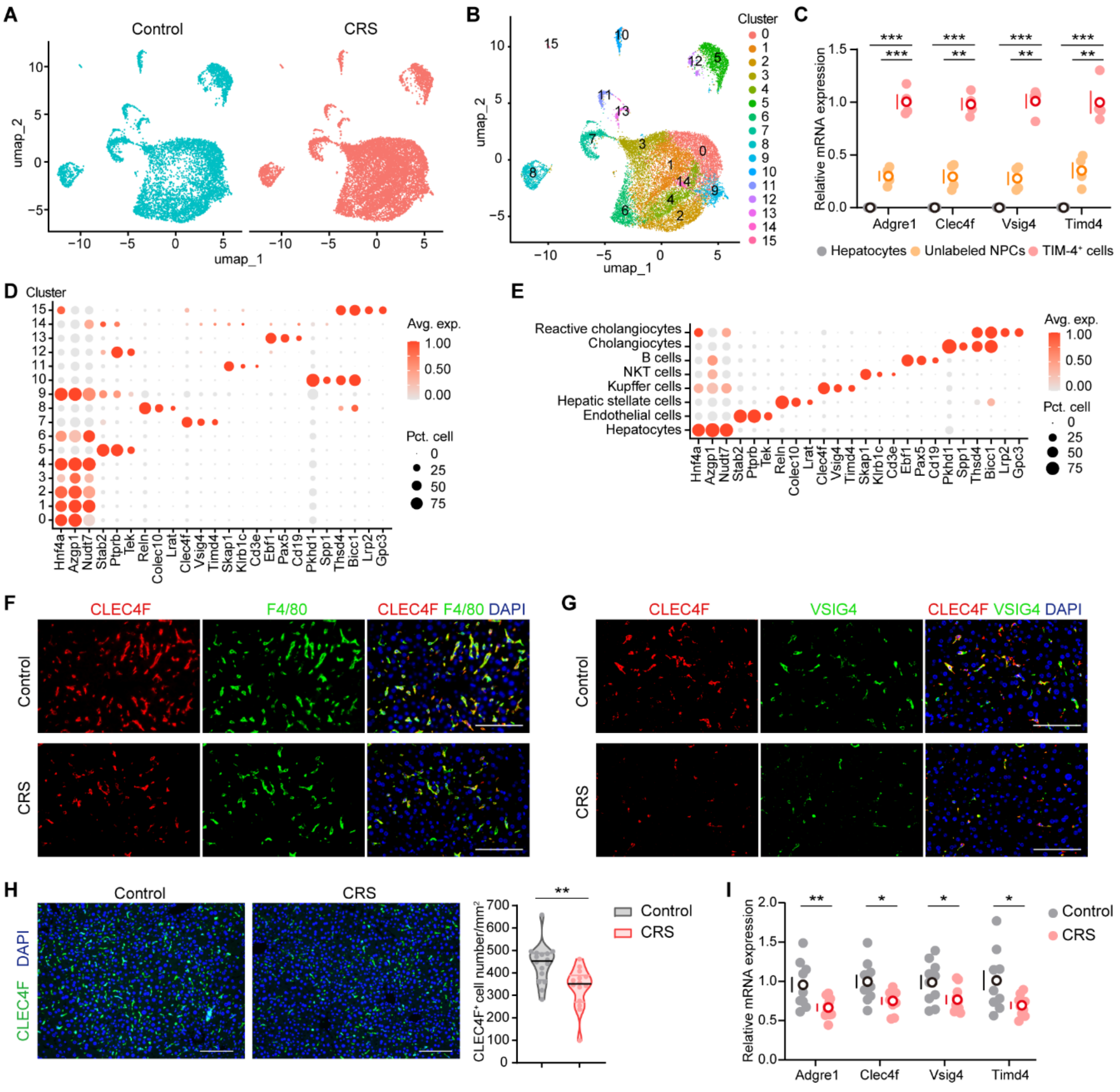

Fig. S2. Single-cell level transcriptome profiling of the liver following chronic stress. (**A** and **B**) UMAP visualization of all cells in control group and CRS group generated by 10× chromium protocol. Colors indicated groups (A) or 16 cell clusters (B). (**C**) qPCR measured the mRNA levels of *Adgre1* (which encodes the macrophage/Kupffer cell marker F4/80), *Clec4f*, *Vsig4*, and *Timd4* (which encodes TIM-4) in hepatocytes, MACS-isolated TIM-4$^+$ cells, and unlabeled NPCs (n = 4). High co-expression of these markers in TIM-4$^+$ cells supports their identity as Kupffer cells. (**D** and **E**) Dot plot showing the scaled expression levels and cellular percentages of feature genes in 16 cell clusters (D) or among cell subpopulations (E). (**F**) Immunofluorescent co-staining of CLEC4F and F4/80. (**G**) Immunofluorescent co-staining of CLEC4F and VSIG4. (**H**) Immunofluorescent staining of CLEC4F and the number of CLEC4F$^+$ cells per mm^2 were quantified (n = 5). The median was indicated. (**I**) qPCR measured the mRNA levels of *Adgre1*, *Clec4f*, *Vsig4*, and *Timd4* (n = 10). Scale bars: 100 µm. Except for H, data are presented as the mean ± SEM; *$P < 0.05$, **$P < 0.01$, ***$P < 0.001$, *t*-test.

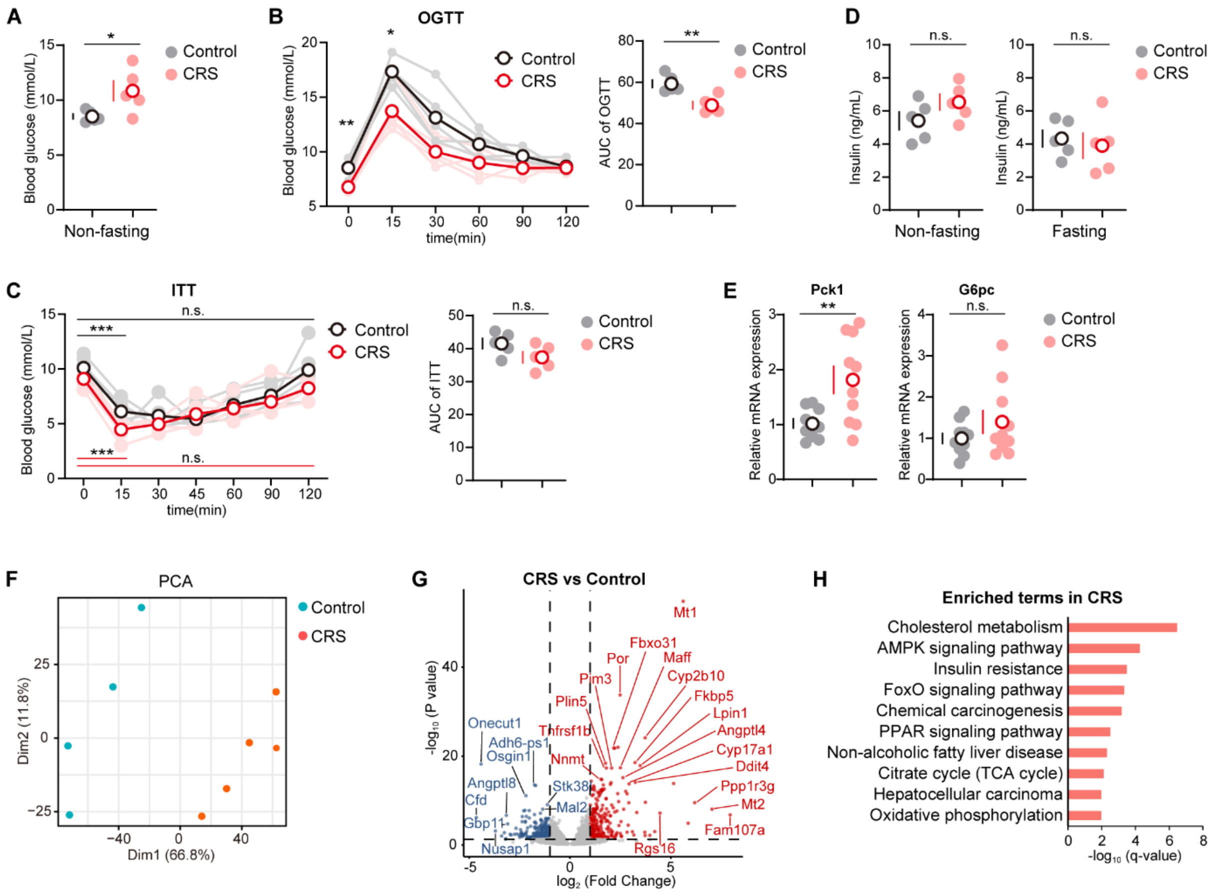

Fig. S3. Chronic stress induces hepatic dysfunction. (**A**) Blood glucose levels in mice under non-fasting condition (n = 5). (**B**) Blood glucose curves (left) and AUC (right) of oral glucose tolerance test (OGTT) in fasted mice (n = 5). (**C**) Blood glucose curves (left) and AUC (right) of insulin tolerance test (ITT) in fasted mice (n = 5). (**D**) Insulin levels in serum under non-fasting and fasting conditions (n = 5). (**E**) qPCR measured the mRNA levels of *Pck1* (a key gluconeogenic gene) and *G6pc* (encodes the final step enzyme in gluconeogenesis) (n = 10). (**F**) Principal component analysis (PCA) of bulk RNA-seq analysis in control and CRS-exposed mouse livers (Control, n = 4; CRS, n = 5). (**G**) Volcano plot showed DEGs in CRS-exposed mouse livers when compared with control livers. (**H**) Enriched KEGG terms in CRS-exposed mouse livers when compared with control livers. Data are presented as the mean ± SEM; n.s., not significant; *$P < 0.05$, **$P < 0.01$, ***$P < 0.001$, *t*-test.

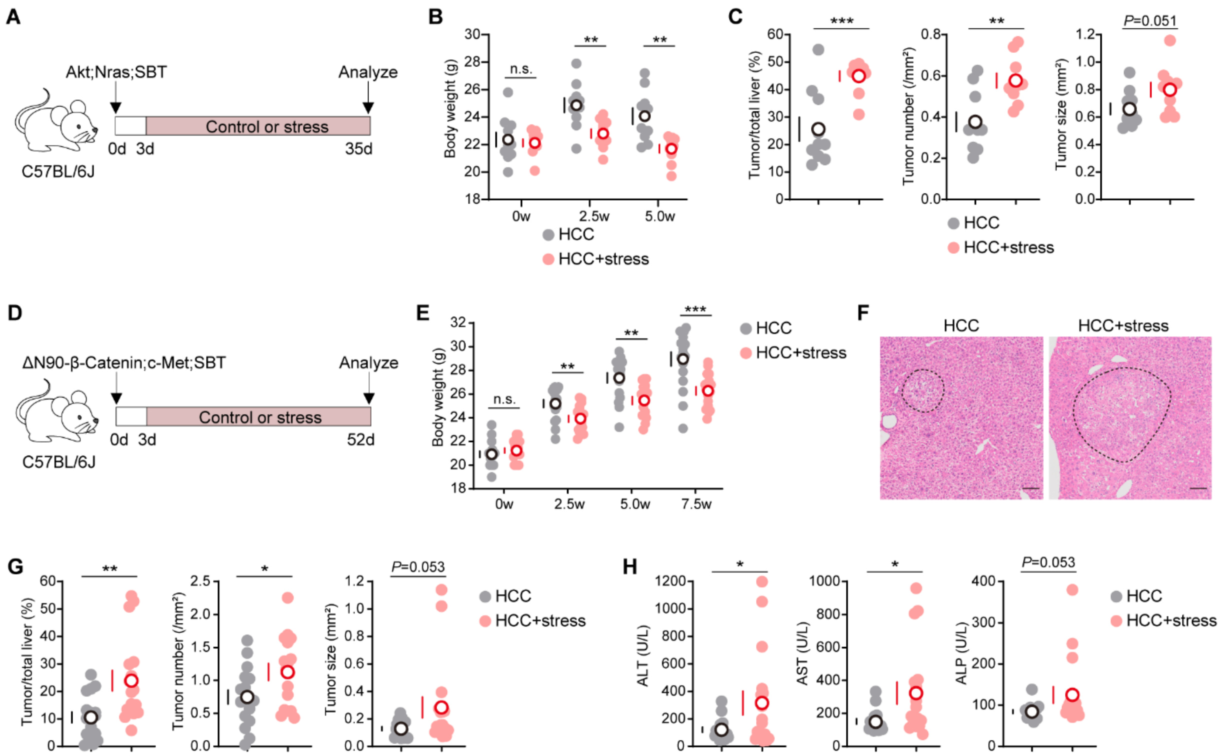

Fig. S4. Chronic stress accelerates liver cancer progression. (**A**) Schematic view of tumor modeling and stress treatment. Liver cancer was induced by HTVi of Akt;Nras;SBT in 8-week-old mice. These mice were then treated with daily stressors or without stress treatment. All mice were sacrificed 35 days later to quantify the tumor formation (B-C; n = 10). (**B**) Body weights. (**C**) The ratios of tumor/total liver area, tumor number per mm^2, and tumor size. (**D**) Schematic view of HCC modeling and stress treatment. HCC was induced by HTVi of ΔN90-β-Catenin;c-Met;SBT in 8-week-old mice. These mice were then treated with daily stressors or without stress treatment. All mice were sacrificed 52 days later to quantify the tumor formation (E-H; n = 17). (**E**) Body weights. (**F**) Representative H&E staining images of liver sections. Tumors were circled by black lines. (**G**) The ratios of tumor/total liver area, tumor number per mm^2, and tumor size. (**H**) Serum levels of ALT, AST, and ALP. Scale bars: 100 μm. Data are presented as the mean ± SEM; n.s., not significant; *$P < 0.05$, **$P < 0.01$, ***$P < 0.001$, *t*-test.

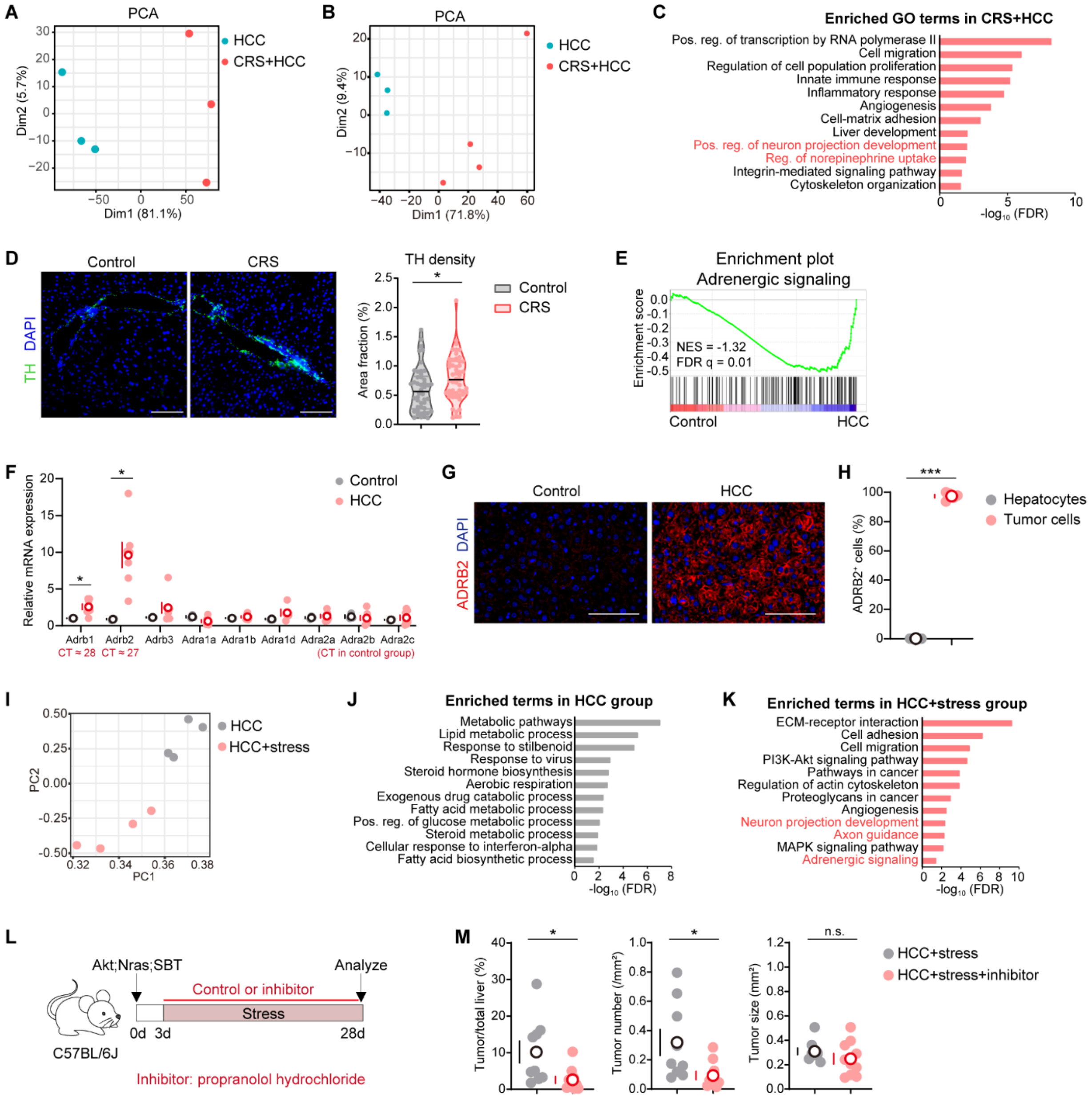

Fig. S5. Adrenergic signaling is involved in liver cancer progression. (**A**) PCA of bulk RNA-seq analysis in CRS+HCC and HCC tissues in Akt;Nras;SBT-induced tumor model (n = 3). (**B** to **H**) Liver cancer model: ΔN90-β-Catenin;c-Met;SBT. (**B**) PCA of bulk RNA-seq analysis in CRS+HCC (n = 4) and HCC tissues (n = 3). (**C**) Enriched GO terms in CRS+HCC tissues when compared with HCC tissues. (**D**) Immunofluorescent staining of the sympathetic nerve marker TH. TH density was quantified (n = 5). The median was indicated. (**E**) The GSEA analysis of the gene set of adrenergic signaling in HCC tissues versus control livers. (**F**) qPCR measured the mRNA levels of nine adrenergic receptor genes (Control, n = 3; HCC, n = 7; For *Adra1d* in HCC, n = 5). (**G**) Immunofluorescent staining of ADRB2. (**H**) The ratio of ADRB2^{+} cells (n = 5). (**I** to **M**) Liver cancer model: Akt;Nras;SBT. (**I**) PCA of bulk RNA-seq analysis in HCC+stress and HCC tissues (n = 4). (**J** and **K**) Enriched GO terms in HCC tissues (J) versus HCC+stress tissues (K). (**L**) Schematic view of tumor modeling, stress treatment, and propranolol treatment. 8-week-old mice

were induced tumor by HTVi of Akt;Nras;SBT. These mice were then treated with daily stressors. Mice were randomly assigned to receive either propranolol or the vehicle control. All mice were sacrificed 4 weeks later to quantify the tumor formation (M; HCC+stress, n = 9; HCC+stress+inhibitor, n = 10). (**M**) The ratios of tumor/total liver area, tumor number per mm^2, and tumor size. Scale bars: 100 μm. Except for D, data are presented as the mean ± SEM; n.s., not significant; *$P < 0.05$, ***$P < 0.001$, *t*-test.

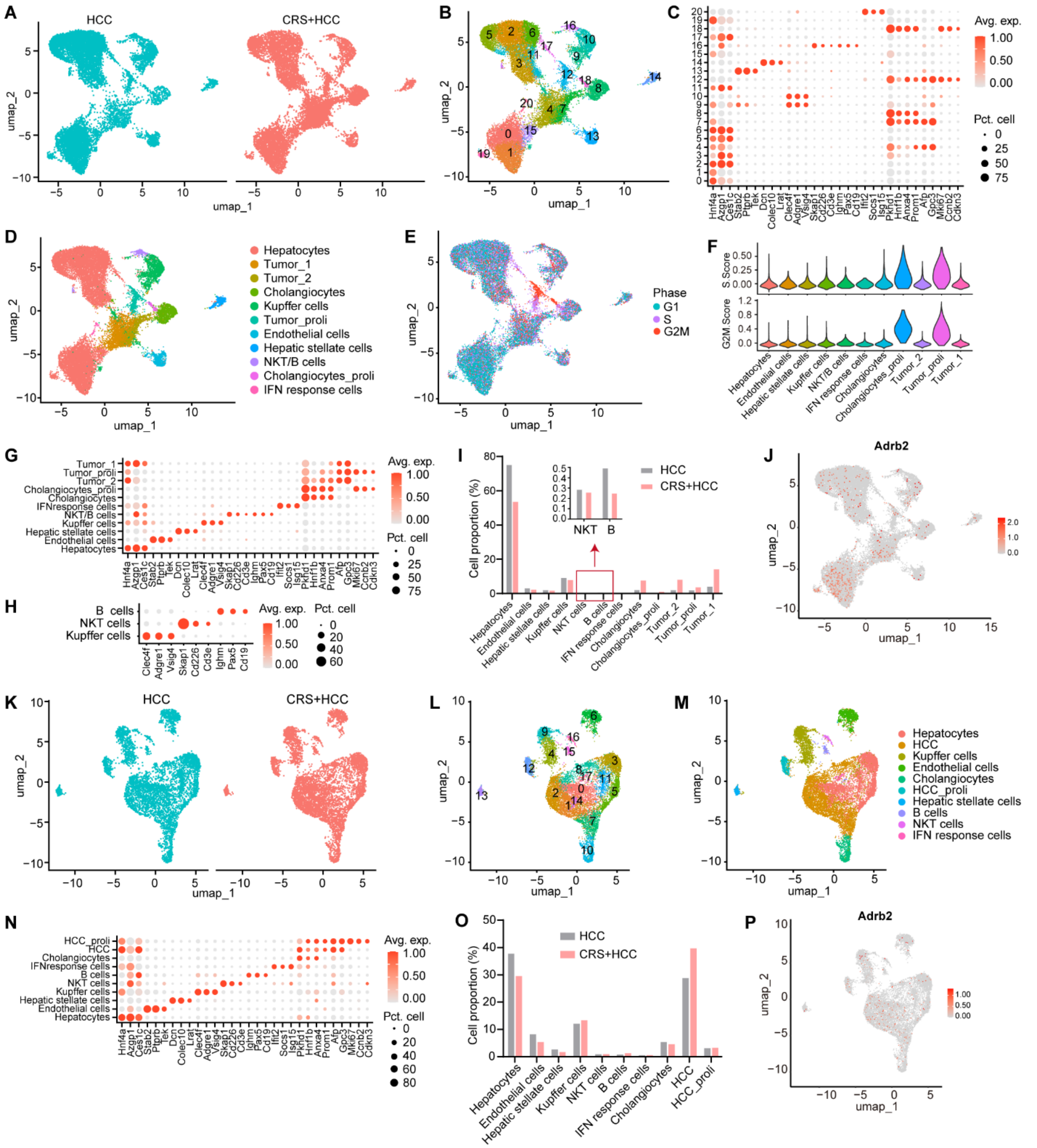

Fig. S6. SnRNA-seq defines the hepatic cellular landscape in tumor-bearing mice with and without chronic stress. (**A** to **J**) Liver cancer model: Akt;Nras;SBT. (**A**) UMAP visualization. Colors indicated groups. (**B**) UMAP visualization. Colors indicated 21 cell clusters. (**C**) Dot plot showing the scaled expression levels and cellular percentages of feature genes in 21 cell clusters. (**D**) UMAP visualization. Colors indicated cell subpopulations. (**E**) UMAP visualization. Colors indicated cell phase. (**F**) Violin plot showing S score levels or G2M score levels in cell subpopulations. (**G** and **H**) Dot plot showing the scaled expression levels and cellular percentages of feature genes among cell subpopulations. (**I**) The cell proportions of different cell

subpopulations in each group. (**J**) UMAP visualization showing the scaled expression levels of *Adrb2*. (**K** to **P**) Liver cancer model: ΔN90-β-Catenin;c-Met;SBT. (**K**) UMAP visualization. Colors indicated groups. (**L**) UMAP visualization. Colors indicated 18 cell clusters. (**M**) UMAP visualization. Colors indicated cell subpopulations. (**N**) Dot plot showing the scaled expression levels and cellular percentages of feature genes among cell subpopulations. (**O**) The cell proportions of different cell subpopulations in each group. (**P**) UMAP visualization showing the scaled expression levels of *Adrb2*.

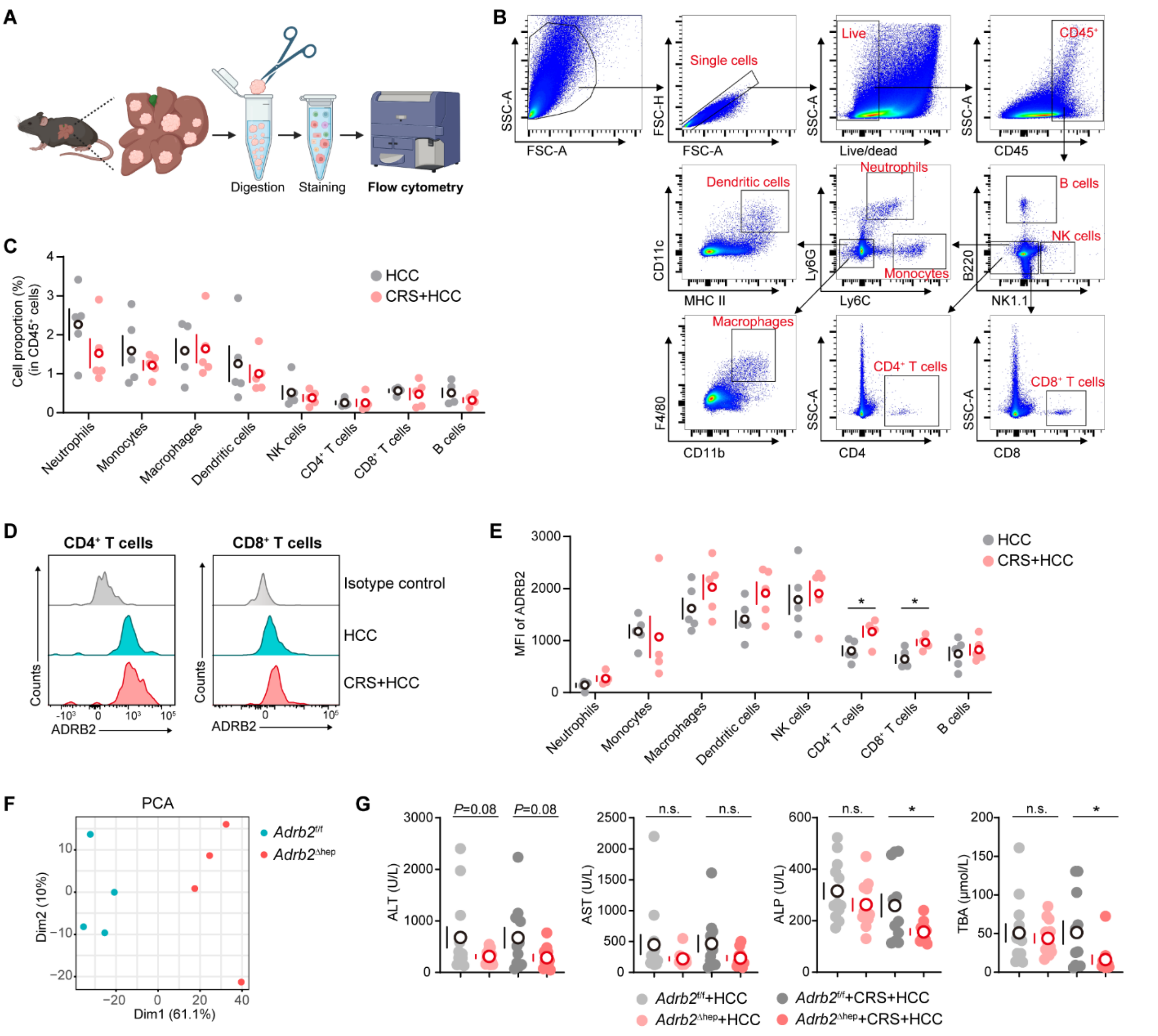

Fig. S7. Flow cytometric analysis of ADRB2 expression in immune cells from liver tumors. (**A**) Schematic overview of the experimental workflow for processing liver tumors to generate single-cell suspensions for flow cytometry. Created in BioRender. Li, L. (2026) https://BioRender.com/jxksvov. (**B**) Gating strategy of flow cytometry for measuring intracellular ADRB2 protein level in immune cells from liver tumors. B cells ($CD45^{+}NK1.1^{-}B220^{+}$), NK cells ($CD45^{+}B220^{-}NK1.1^{+}$), $CD4^{+}$ T cells ($CD45^{+}NK1.1^{-}B220^{-}CD4^{+}$), $CD8^{+}$ T cells ($CD45^{+}NK1.1^{-}B220^{-}CD8^{+}$), monocytes ($CD45^{+}NK1.1^{-}B220^{-}Ly6G^{-}Ly6C^{+}$), neutrophils ($CD45^{+}NK1.1^{-}B220^{-}Ly6C^{-}Ly6G^{+}$), macrophages ($CD45^{+}NK1.1^{-}B220^{-}Ly6G^{-}Ly6C^{-}F4/80^{+}CD11b^{+}$), and dendritic cells ($CD45^{+}NK1.1^{-}B220^{-}Ly6G^{-}Ly6C^{-}MHCII^{+}CD11c^{+}$) were characterized by specific surface markers. (**C**) The cell proportions of different cell populations (n = 5). (**D**) Representative flow cytometric analysis of ADRB2 expression in $CD4^{+}$ T cells and $CD8^{+}$ T cells. (**E**) ADRB2 protein levels in different cell populations (n = 5). MFI, mean fluorescence intensity. (**F**) PCA of bulk RNA-seq analysis in *Adrb2*$^{f/f}$ and *Adrb2*$^{\Delta li}$ hepatocytes. (**G**) Serum levels of ALT, AST, ALP, and TBA. Data are presented as the mean ± SEM; n.s., not significant; *$P < 0.05$, *t*-test.

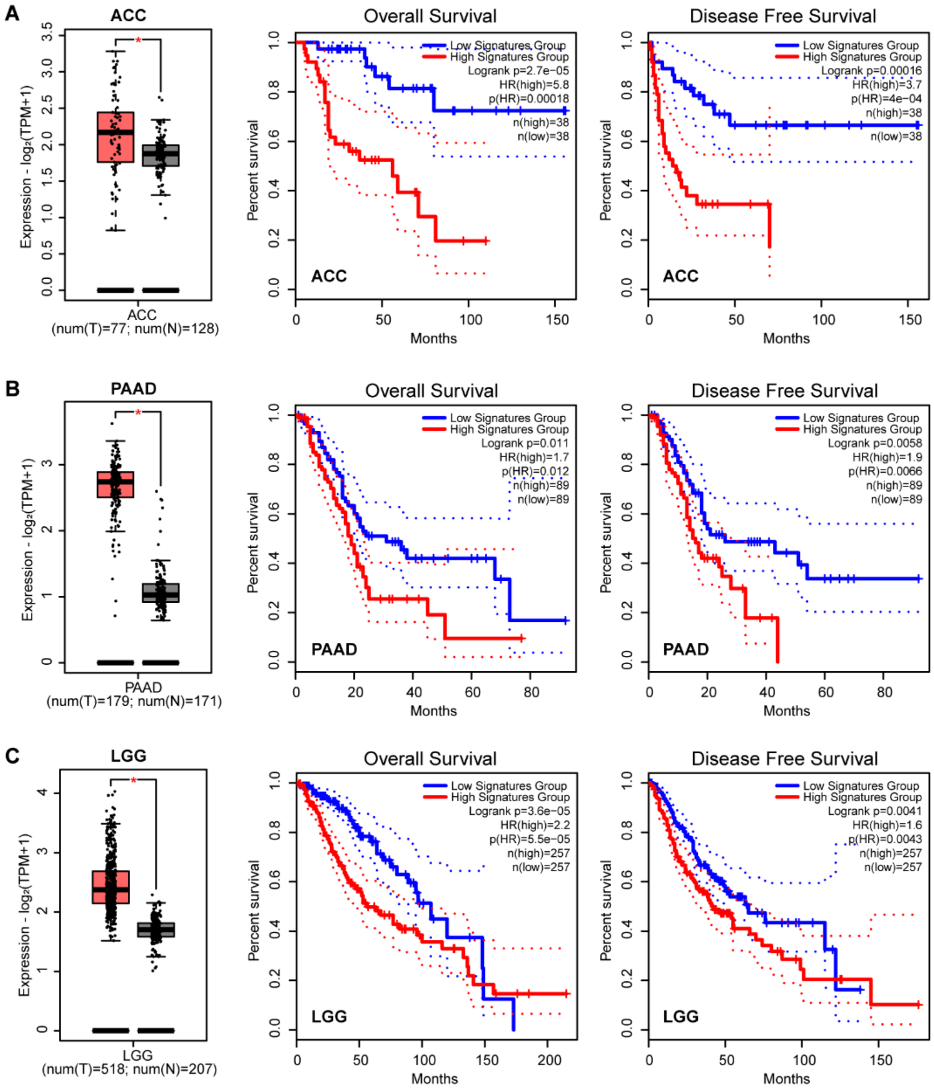

Fig. S8. Elevated ADRB2 signaling is associated with poor clinical outcomes in cancer patients. (**A** to **C**) The boxplot (left) showed the expression levels of ADRB2 signaling genes in TCGA and GTEx normal tissues (gray) and TCGA tumor tissues (red). Based on the overall expression of ADRB2 signaling genes in tumor tissues, patients were classified into the high signature group (top 50%) and the low signature group (bottom 50%). Kaplan-Meier analysis showed the overall survival (middle) and disease-free survival (right) of patients. ACC, adrenocortical carcinoma. PAAD, pancreatic adenocarcinoma. LGG, brain lower grade glioma.

Table S1. (separate file)
Gene sets associated with HCC progression, NAFLD, adrenergic and ADRB2 signaling

Table S2. (separate file)
Primers for qPCR

Data file S1. (separate file)
Source data for experiments with sample size $n < 20$
